# Operating regimes in a single enzymatic cascade at ensemble-level

**DOI:** 10.1371/journal.pone.0220243

**Published:** 2019-08-01

**Authors:** Akshay Parundekar, Girija Kalantre, Akshada Khadpekar, Ganesh A. Viswanathan

**Affiliations:** Department of Chemical Engineering, Indian Institute of Technology Bombay, Powai, Mumbai, India; Bose Institute, INDIA

## Abstract

Single enzymatic cascade, ubiquitously found in cellular signaling networks, is a phosphorylation-dephosphorylation reaction cycle causing a transition between inactive and active states of a protein catalysed by kinase and phosphatase, respectively. Steady-state information processing ability of such a cycle (e.g., MAPK cascade) has been classified into four qualitatively different operating regimes, *viz*., hyperbolic (H), signal-transducing (ST), threshold-hyperbolic (TH) and ultrasensitive (U). These four regimes represent qualitatively different dose-response curves, that is, relationship between concentrations of input kinase (e.g., pMEK) and response activated protein (e.g., pERK). Regimes were identified using a deterministic model accounting for population-averaged behavior only. Operating regimes can be strongly influenced by the inherently present cell-to-cell variability in an ensemble of cells which is captured in the form of pMEK and pERK distributions using reporter-based single-cell experimentation. In this study, we show that such experimentally acquired snapshot pMEK and pERK distribution data of a single MAPK cascade can be directly used to infer the underlying operating regime even in the absence of a dose-response curve. This deduction is possible primarily due to the presence of a monotonic relationship between experimental observables R_IQR_, ratio of the inter-quartile range of the pERK and pMEK distribution pairs and R_M_, ratio of the medians of the distribution pair. We demonstrate this relationship by systematic analysis of a quasi-steady state approximated model superimposed with an input gamma distribution constrained by the stimulus strength specific pMEK distribution measured on Jurkat-T cells stimulated with PMA. As a first, we show that introduction of cell-to-cell variability only in the upstream kinase achieved by superimposition of an appropriate input pMEK distribution on the dose-response curve can predict bimodal response pERK distribution in ST regime. Implementation of the proposed method on the input-response distribution pair obtained in stimulated Jurkat-T cells revealed that while low-dosage PMA stimulation preserves the H regime observed in resting cells, high-dosage causes H to ST regime transition.

## Introduction

Single enzymatic cascade or a phosphorylation-dephosphorylation reaction cycle, is a crucial, ubiquitously conserved, building-block in a cellular signaling network [1,2]. Single MAPK cascade acts as signal amplifier and its aberrant functioning has been implicated in many diseases such as cancer [3,4]. Detailed understanding of the behavior of such a cascade can offer useful insights to design therapeutic strategies for combating certain diseases.

**Fig 1:**
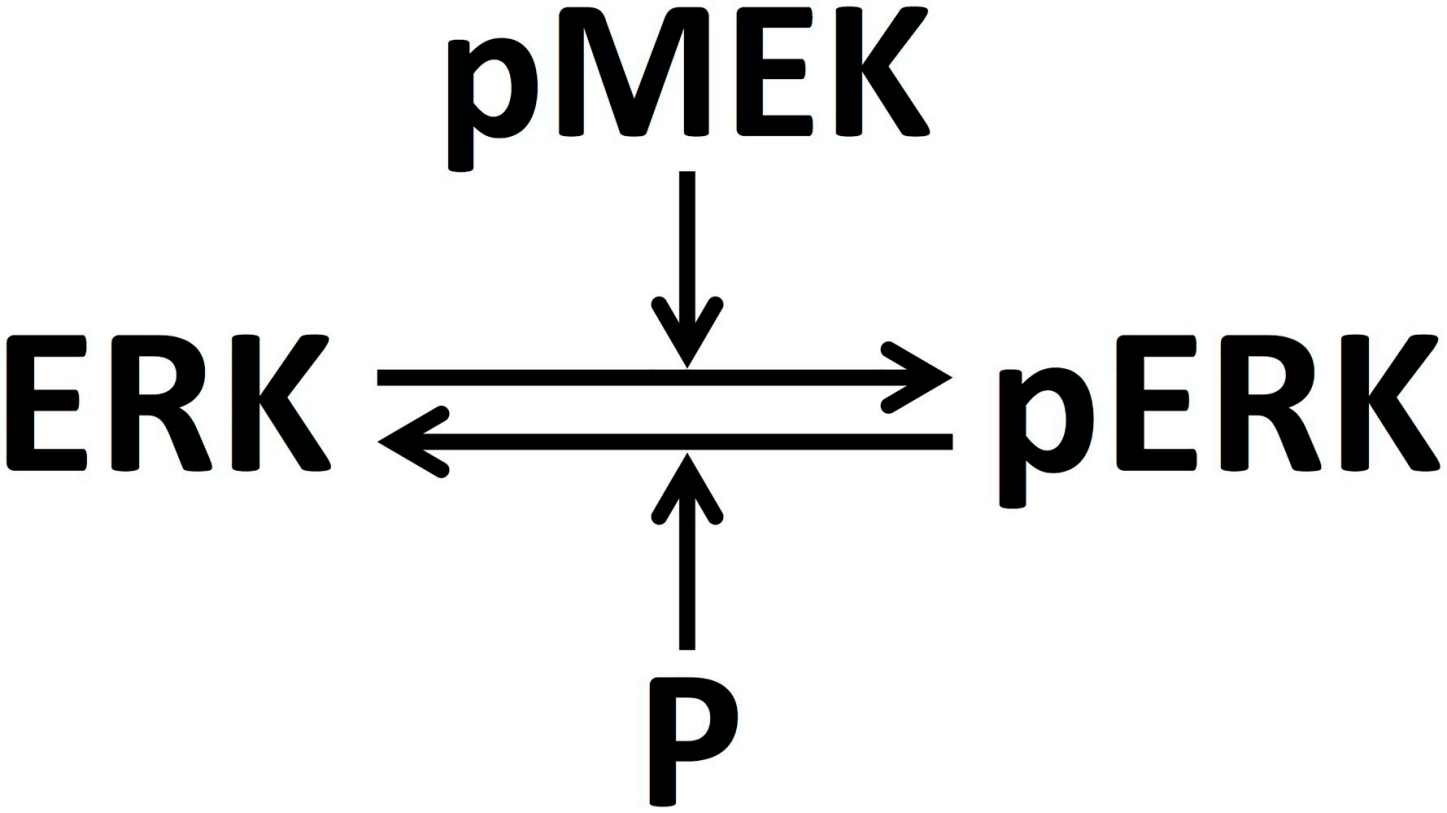
Schematic of a single MAPK enzymatic cascade.

In the phosphorylation reaction of a single MAPK cascade, transition of inactive form of a protein (unphosphorylated ERK) to its active form (phosphorylated ERK or pERK) is catalysed by an input kinase (phosphorylated MEK or pMEK) (Fig 1). On the other hand, a phosphatase (P) enzymatically deactivates pERK via a transition from active to inactive form in the dephosphorylation reaction (Fig 1). Response of a cell exposed to stimulus of a certain strength is known to be governed by the concentration of the input kinase and that of the activated protein (output) of such cascades [5]. An enzymatic reaction (in the cascade) is considered (a) *saturated* if all of the corresponding enzyme is bound to its substrate and (b) *unsaturated* otherwise. Dose-response curve, that is, input-output characteristic of the cycle at steady-state, corresponding to each of the four combinations of saturated/unsaturated behavior of the two enzymatic reactions has been shown to be distinct [6]. These four distinct classes of dose-response curves (or operating regimes) correspond to hyperbolic, signal-transducing, threshold-hyperbolic and ultrasensitive behaviors [6,7]. Such a classification is arrived at using deterministic framework wherein concentration of any protein is assumed equal in all cells in a population. Thus, identification of the regimes from experimental data is currently amenable to only population-averaged measurements that corresponds to deterministic framework. However, it is well established that cell-to-cell variability in the proteins involved in MAPK cascade can strongly impact cellular decision-making process resulting in a certain phenotype (or eventual response) [8,9]. This variability could influence the underlying operating regime that governs the protein abundance in an individual cell. Recent experimental techniques permit precise measurement of abundance of MAPK proteins, both phosphorylated and total, in an ensemble of cells [10]. Therefore, it is necessary to find the operating regimes based on protein abundance information available at the ensemble-level.

The deterministic framework based operating regimes of a single MAPK cascade were identified by contrasting the total quasi-steady state approximation (tQSSA) model guided dose-response curves for a specific set of parameters with nominal profiles corresponding to the four distinct regimes [6]. The nominal profile for a regime was arrived at by incorporating the saturated/unsaturated state of the two enzymatic reactions in the corresponding rates in the model. Moreover, operating regimes have been shown to be sensitive to the concentrations of the kinase and phosphatase [11–14]. Attempts to predict the experimentally observed dose-response curve for MAPK cascade [9,15] using deterministic modeling framework were based on steepness of the input-output behavior [7,16], constraints on the availability of kinase [12], variation in the effective stimulus strength [17].

Simultaneous detection of the abundance of activated proteins such as pMEK and pERK in a single MAPK enzymatic cascade, at an ensemble level is typically achieved by quantitatively capturing fluorescence emitted by an appropriate (non-plasmid) reporter such as an antibody [5,9,15,18]. These reporter-based detection techniques hinge on arresting the biochemical reactions and thereby preserving the instantaneous state of the cell (cell-fixation) and on cell membrane permeabilization for permitting entry of protein specific antibodies. As a result, they are suitable for obtaining snapshots of the distribution of abundance of activated proteins at the discrete sampling points [19] and not amenable for capturing continuous time-trajectories of ensemble of cells. When the pMEK and pERK abundance in a population of cells reach a steady-state, can the underlying operating regime of the MAPK cascade be directly inferred from such ensemble-level snapshot distributions of these two proteins?

In order to address this question, using Michaelis-Menten approximated mathematical model of the single MAPK cascade, we systematically assess the nature of relationship between representative signatures such as inter-quartile range of the distributions of pMEK and of pERK. We incorporated cell-to-cell variability in the model by superimposing the properties of experimentally measured pMEK snapshot distribution in Jurkat E6.1 cells stimulated with different PMA concentrations under steady-state conditions. We show that a monotonicity property in the relationship between two specific signatures of the pMEK and pERK snapshot distributions can be reliably capitalized upon for identifying the operating regimes. Using this relationship, we suggest the regime in which the cascade is operating as dictated by the measured experimental distributions.

## Results

We first consider single-cell level measurement of pMEK and pERK in Jurkat T-cells stimulated with Phorbol Myristate Acetate (PMA). Stimulation of cells with PMA is a standard to study steady-state and dynamical behavior of MAPK cascades in many cellular systems such as T-cells [20–24].

We considered three treatment conditions, *viz*., 1 ng/ml (T1), 100 ng/ml (T2), 1000 ng/ml (T3) PMA and six exposure times, *viz*., 0 (unstimulated or control), 6, 12, 18, 24, 30 mins. Using a dual-staining flow cytometry assay (Materials and methods), we simultaneously detected the instantaneous snapshots of fluorescence levels of pMEK and pERK in a population of cells for each of the 48 (3 treatments x 5 time points x 3 replicates + 1 unstimulated x 3 replicates) experimental samples. (Details of data availability are S1 Text) Instrument and handling variability were minimized by employing a high-throughput fluorescent cell barcoding (FCB) technique before staining and acquisition [25] (Materials and methods).

**Fig 2:**
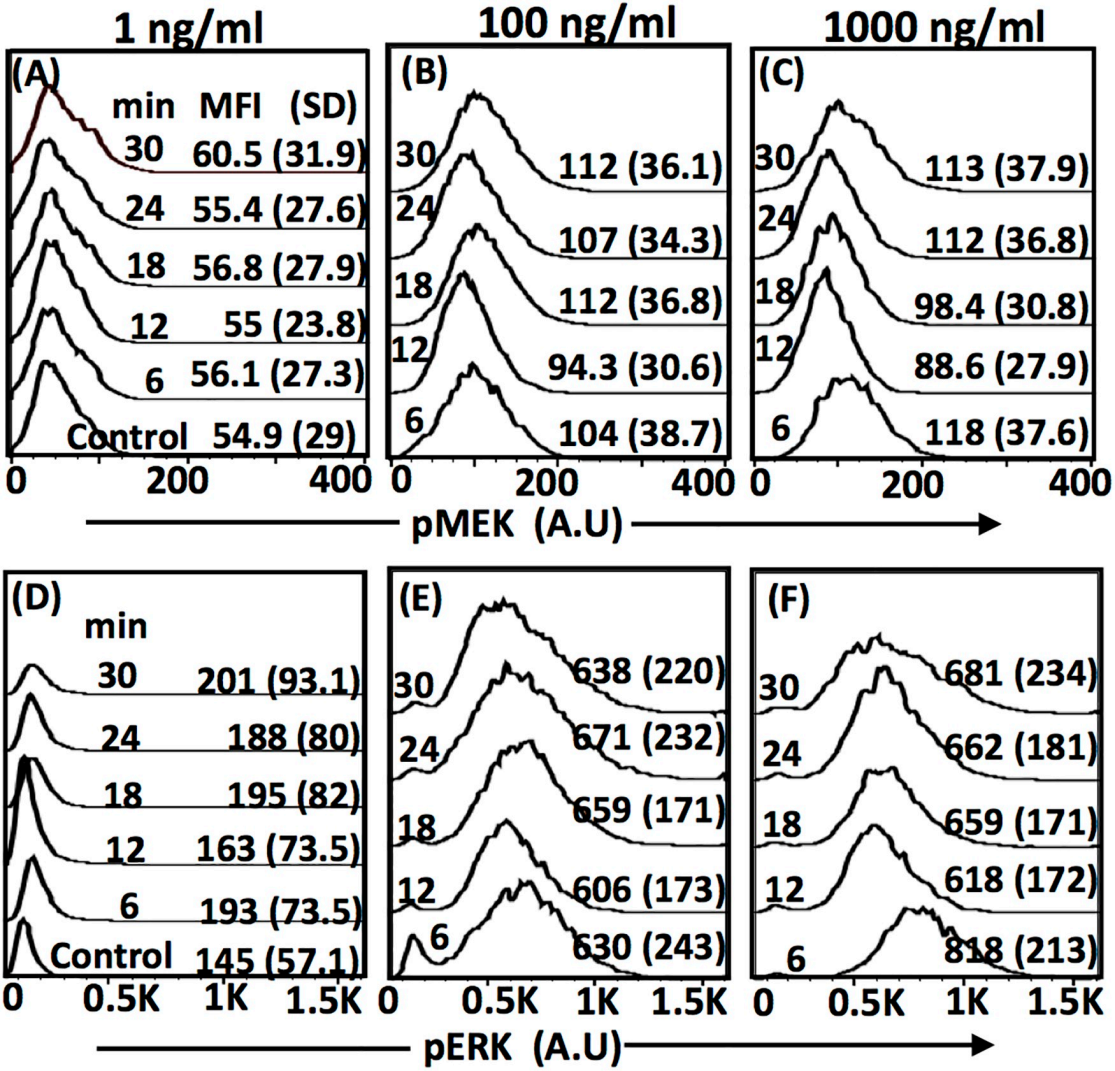
Normalized distributions of pMEK and pERK in Jurkat E6.1 cells exposed to 1 ng/ml (A & D), 100 ng/ml (B & E), 1000 ng/ml (C & F) PMA for different durations. The cells were dual-stained with Alexa488-pERK(1/2) and Alexa647-pMEK. Distribution corresponding to the control (unstimulated) is in the panel for 1 ng/ml (A & D). MFI and SD, respectively for every distribution corresponds to its mean fluorescence intensity and the standard deviation. Replicates are in S1 Fig.

For the three treatment conditions, normalized histograms and their corresponding mean fluorescence intensities (MFIs) for both pMEK and pERK for one replicate are in Fig 2. Distributions corresponding to unstimulated (control) for pMEK and pERK, respectively are in Fig 2A (control) and Fig 2D (control). Histograms for the other two replicates are in S1 Fig, Panels I and II. (We note that one of replicates for 100 ng/ml (S1 Fig, Panel II) had ~18% higher cell loss leading to lower cell-count causing the observed deviation, particularly in pERK response). The standard deviation in MFI across the replicates for each of the treatment condition and exposure time combination is in the brackets next to MFI values in Fig 2. While pMEK levels for T1 is similar to that of control (Fig 2A), there is significant activation for treatments T2 and T3. Dynamics of the distributions, MFI and SD (discounted for deviation across replicates) suggests that cells may have reached a stationary-state within 30 mins for all three treatments. Thus, henceforth, for the rest of the results reported, we assume the pMEK and pERK snapshot distributions obtained at 30 mins correspond steady-state ensemble-level response for each of the three treatment conditions. (In the last part of this section, using stochastic simulations, we show that single enzymatic cascade reaching steady-state in 30 mins is indeed feasible).

### Mathematical model: Ensemble-level behavior

The biochemical reactions corresponding to the single-enzymatic cascade (Fig 1) are

pMEK+ERK⇌pMEK.ERK→pERK+pMEK (1)

P+pERK⇌P.ERK→ERK+P (2)

where *pMEK*.*ERK* and *P*.*pERK* are the intermediate species representing enzyme being bound to its substrate. Following Michaelis-Menten approximation, we assume quasi-steady state for these two intermediate species. Mathematical model capturing the dynamics of the concentration of pERK, *M*_*p*_, permitted by the biochemical reactions in Eqs 1 and 2 is

$$\frac{dM_{p}}{dt}=\frac{k_{f}E(M_{t}-M_{p})}{K_{1}+(M_{t}-M_{p})}-\frac{k_{r}pM_{p}}{K_{2}+M_{p}}$$ (3)

where, *k*_*f*_ and *k*_*r*_ are the forward and reverse catalytic rate constants. While *K*_*1*_ and *K*_*2*_ are Michaelis-Menten constants, *p*, *E*, and *M*_*t*_, respectively are the concentrations of P, pMEK and total ERK [7,26]. At steady-state, the feasible analytical solution of Eqs 1 and 2 is

$$\frac{M_{p}}{M_{t}}=\frac{-b+\sqrt{b^{2}-4(k_{f}E/k_{r}p)(1-k_{f}E/k_{r}p)(K_{2}/M_{t})}}{2(k_{f}E/k_{r}p-1)}$$ (4)

where, *b* = −(*k*_*f*_ *E* / *k*_*r*_*p* − 1) + (*K*_2_ / *M*_*t*_)(*k*_*f*_*E* / *k*_*r*_*p*) + (*K*_1_ / *M*_*t*_). The dose-response curve (*M*_*p*_ vs *E*) for a certain set of parameters can be obtained from Eq 4. For the sake of brevity, we henceforth refer to the four operating regimes, *viz*., Hyperbolic, Signal-transducing, Threshold-hyperbolic, and Ultrasensitive, respectively as H, ST, TH, and U.

While regime identification is traditionally achieved by comparing parameter-specific dose-response curve with nominal profiles of H, ST, TH and U ([6], Materials and methods), such an approach is not directly amenable for use when only snapshot data such as those in Fig 2 is available. This is primarily due to the fact that for such kind of experimental data the underlying dose-response curve is typically unavailable. Therefore we consider identification of operating regime by contrasting the steady-state snapshot distributions of pMEK (input) and pERK (response). In the following sections, we first present a qualitative assessment of the operating regimes when ensemble-level features are incorporated and subsequently decipher the monotonicity feature between experimental observables that can be capitalized upon for finding the regime permitted by an input and response distribution pair.

The parameters used for the model simulations are in Table 1. Note that values for *k*_*f*_, *k*_*r*_, *p* and *M*_*t*_ were kept constant for all simulations. *K*_*1*_ and *K*_*2*_ combination for the four distinct operating regimes reported in Table 1 were used to obtain the nominal H, ST, TH, and U profiles [6]. Further, a sampling strategy (Materials and methods) was used to generate 140000 (*K*_1_, *K*_2_) sample sets. Dose-response curve corresponding to each of the (*K*_1_, *K*_2_) samples were contrasted with nominal profiles of the four operating regimes. The samples belonging to four different regimes were identified (Materials and methods). While 58.9% of (*K*_1_, *K*_2_) sets were placed in H, ST, TH, and U, the dose-response curves corresponding to the remaining samples do not satisfy the regime-identification criteria. For the rest of this study, only those (*K*_1_, *K*_2_) sets that satisfied the regime-identification criteria were used for further analysis. Note that the remaining 41.1% of the (*K*_1_, *K*_2_) samples would fall into input-output behavior other than these four regimes. Some of these behaviors that require consideration of variability in other parameters for systematic characterization have been considered at deterministic level by Straube [11]. The goal of the current study is restricted to those dose response behaviors corresponding to the saturated/unsaturated state of the two enzymatic reactions achieved by constraining (*K*_1_, *K*_2_), we restrict the analysis to the corresponding four operating regimes only.

**Table 1:** Parameters corresponding to nominal profile for the four operating regimes. These are based on those reported in Gomez-Uribe et al [6].

Regime/ Parameter	*k*_*f*_ (s^-1^)	*k*_*r*_ (s^-1^)	*K*_*1*_ (nM)	*K*_*2*_ (nM)	*p* (nM)	*M*_*t*_ (nM)
**Hyperbolic**	0.01	0.01	10000	10000	200	1000
**Signal-transducing**	0.01	0.01	10	10000	200	1000
**Threshold-Hyperbolic**	0.01	0.01	10000	1	200	1000
**Saturated**	0.01	0.01	10	10	200	1000

### Bimodal response in signal-transducing regime

In the deterministic framework, input-output relationship for any (*K*_1_, *K*_2_) within a regime will be qualitatively similar. This is due to the fact that all dose-response curves in a regime is contrasted against the same pre-set nominal profile. However, this may not be the case when distribution features representing ensemble-level behavior are considered [5,18]. The ensemble-level behavior of a single enzymatic cascade typically entails characteristics of the input and the response distributions, each of which can permit different shapes. Qian et al. suggest that stochastic bifurcations leading to bimodal response of a single enzymatic cascade may be possible under the assumption that both upstream kinase and phosphatase are fluctuating simultaneously with sum of its variances being sufficiently large [13]. These constraints may not be valid in all operating regimes. Moreover, in the pMEK-pERK system, while the kinase level is dictated by the upstream signaling machinery, the phosphatase not necessarily so. We ask a question can the response distributions at different (*K*_1_, *K*_2_) set from the same operating regime exhibit qualitatively different shape such as unimodal, bimodal in spite of input distributions being similar while assuming phosphatase is maintained constant. In order to answer this question, we systematically distill out possible response distribution shapes within each of the four operating regimes by assuming a specific source for cell-to-cell variability.

**Fig 3:**
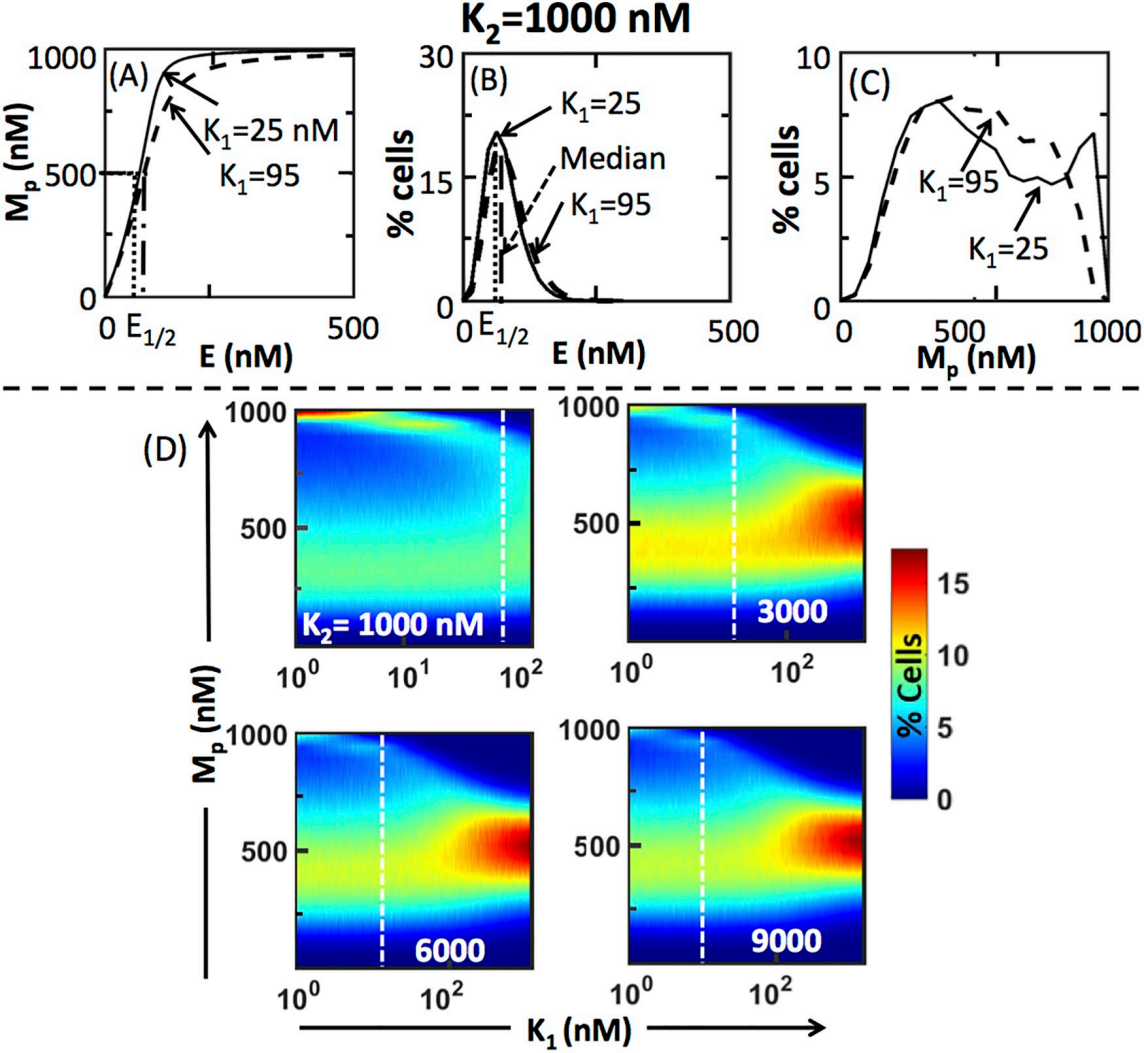
Signal-transducing regime can permit both unimodal and bimodal response. (A) Dose-response curves corresponding to (*K*_*1*_,*K*_*2*_) = (25,1000) and (95,1000), both in ST regime. (B) Input kinase distribution (with E_1/2_ as median and shape parameter of 4.35) corresponding those profiles in (A). (C) The response distribution corresponding to the profiles in (A) subjected to the input kinase distribution in (B). (D) Normalized pERK distributions in the ST regime in planes of *K*_*1*_ and *M*_*p*_ for four different fixed *K*_*2*_. White dashed line separates the (*K*_*1*_, *M*_*p*_) plane based on unimodal or bimodal nature of the normalized response distribution (*M*_*p*_) using Hartigan’s dip test (Materials and methods).

Variability in the response of a population of cells can be attributed to several sources such as distribution of protein levels in resting cells, variation in cell-cycle stages [27,28]. In this study, we assume that the variability across cells in a population stems from the possibility of levels of upstream enzyme pMEK being different in them, as suggested in a few recent studies [13,17,29]. Under these assumptions, it is known that dose-response curves for a certain (*K*_1_, *K*_2_) in TH [18] and U [9,13] regimes may result in bimodal pERK distribution when the upstream kinase experiences a slow dynamic fluctuations (with phosphatase not fluctuating). Further, using the truncated geometric distribution [30], for the U regime where both enzymatic reactions follow zero-order kinetics, Qian et al. [13] showed that slow fluctuations in the upstream kinase *alone* is sufficient for obtaining a bimodal distribution in the active form of the substrate. However, it is as yet unclear if the dose-response curves in the ST regime could also permit a bimodal response behavior when variability is present only in the upstream kinase. In order to assess this possibility, we first consider two distinct dose-response curves (obtained using Eq 4) in the ST regime, *viz*., for *K*_*1*_ = 25 nM and *K*_*1*_ = 95 nM for a fixed *K*_*2*_ = 1000 nM (Fig 3A). Note that in ST regime, phosphorylation and dephosphorylation reactions are in saturated and unsaturated states, respectively. We introduce the cell-to-cell variability into these two dose-response curves by superimposing an input distribution corresponding to steady-state pMEK snapshot normalized histogram reported in Fig 2.

Based on the approach in Birtwistle *et al*. [18], we show in S3 Text that the experimental steady-state pMEK normalized histogram (30 mins sample) in Fig 2 and S1 Fig can be approximated to gamma distribution for unstimulated and all three treatment conditions. Gamma distribution is completely specified using two parameters, *viz*., shape and scale. While the shape parameter uniquely specifies the CV of a gamma distribution, the mean is given by the product of shape and scale parameters. Shape parameter corresponding to the experimental data (including all replicates) were in the range (4.16, 8.65) (S3 Text). We therefore assumed gamma distribution *D*(*E*) for pMEK with a nominal value of shape parameter of 4.35. We later show the effect of shape parameter on the predictions. In order to ensure a poised representation of the entire concentration range of pMEK, we placed the median of *D*(*E*) at *E*_1/2_, the concentration of pMEK corresponding to *M*_*p*_ = 500 nM, that is, half-maximal, for the specified parameters. (Note that *E*_1/2_ for a set of parameters can be uniquely estimated from Eq 4 for a given *M*_*p*_. Moreover, we show in S4 Text that the maximum *M*_*p*_ permitted by Eq 4 will be equal to *M*_*t*_ (= 1000 nM) for sufficiently large *E* irrespective of the other parameters in the model). Using the relationship between the two parameters of gamma distribution and its median (S3 Text), for a fixed shape parameter, we estimated the scale parameter. *D*(*E*) corresponding to the dose response curves at *K*_*1*_ = 25 nM and *K*_*1*_ = 95 nM for a fixed *K*_*2*_ = 1000 nM are in Fig 3B.

We generated 20000 values of *E* from *D*(*E*) specified by the chosen (experimental data guided) shape and scale parameters. For each of these 20000 *E* values, using the dose-response curve for *K*_*1*_ = 25 nM or 95 nM as a map, we found the corresponding *M*_*p*_ level. Thus, by superimposing the corresponding assumed *D*(*E*) on the dose-response curves, we generated the pERK response (*M*_*p*_) distribution for the two cases considered (Fig 3C). In order to assess if the response distribution possesses more than one high point (or mode), we subjected the normalized pERK distributions to Hartigan’s dip test [31] (Materials and methods). The dip test showed that the distribution obtained for *K*_*1*_ = 95 nM is unimodal and that for *K*_*1*_ = 25 nM is not. The response distribution for *K*_1_ = 25 nM is bimodal (Fig 3C). Thus, qualitatively different ensemble-level behavior is possible by varying *K*_1_ for a fixed *K*_2_ within the ST regime.

We next assessed the dependence of the presence of unimodal and bimodal pERK distributions on *K*_1_ with fixed *K*_2_ in the ST regime. In Fig 3D, we show the effect of *K*_*1*_ on the *M*_*p*_ distributions for different fixed *K*_*2*_. For every *K*_1_, the percentage of cells with a certain *M*_*p*_ in the response distribution is captured by the color in the heatmap. Hartigan’s dip test (with p-value < 0.05) was used to separate the *K*_*1*_ parameter space into ranges that permit bimodal and unimodal distributions (dashed white line in Fig 3D). Fig 3D clearly shows that for a wide range of K_2_, there exists two regions in the *K*_1_ parameter space within the ST regime exhibiting characteristically different ensemble-level behavior. Moreover, the range in *K*_*1*_ for which bimodal response distribution may be observed reduced with increase in *K*_*2*_. Similarly, for a certain fixed *K*_1_, the *K*_2_ parameter space can also be separated into two regions in which the response distribution is unimodal and bimodal, respectively. For e.g., when *K*_1_ = 50nM, *M*_*p*_ distribution is bimodal for *K*_2_ of 1000nM (Fig 3D-i) and 3000nM (Fig 3D-ii), and unimodal for *K*_2_ of 6000nM (Fig 3D-iii) and 9000nM (Fig 3D-iv). Since the response distribution could depend on the parameters of *D*(*E*), we show that for a wide range of shape parameters, the ST regime permits both unimodal and bimodal response distributions (S2 Fig).

We next characterised the response distributions for H (S3 Fig), TH (S4 Fig) and U (S5 Fig) regimes. While for the case of TH, both unimodal and bimodal responses were observed at different *K*_*1*_ and *K*_*2*_ ranges, H regime permits only unimodal distribution for all parameter ranges (including shape parameter of the input distribution). Note that we find only bimodal response in U regime. While low values of shape parameter permits only bimodal response in the TH regime, only unimodal response is exhibited for large values of shape parameter (S4 Fig).

### Monotonic relationship between inter-quartile range ratio R_IQR_ and median ratio R_M_

We next consider an approach for regime identification at ensemble-level using snapshot input-response distribution pair. Since dose-response curve depends both on the levels of the input kinase pMEK and that of the response protein pERK, properties of both distributions are considered in arriving at an appropriate approach. Besides the mean and median, which reflect the average properties of a distribution, metrics that capture the variation in the protein levels across cells in a population include variance and coefficient of variation (CV = standard deviation/mean). Since CV of *D*(*E*) is uniquely specified by the shape parameter (S3 Text), it cannot be used as a metric for contrasting the two distributions. On the other hand, given that *D*(*E*) is skewed, we choose inter-quartile range (IQR), a balanced scale estimator [32], of the input (pMEK) and the response (pERK) distributions as representative signature for variability in the respective protein levels. We consider ratio of the IQR of the response and the input distributions, R_IQR_, an experimental observable, which can offer insights on the extent of deviation in the variability offered by the enzymatic cascade.

**Fig 4:**
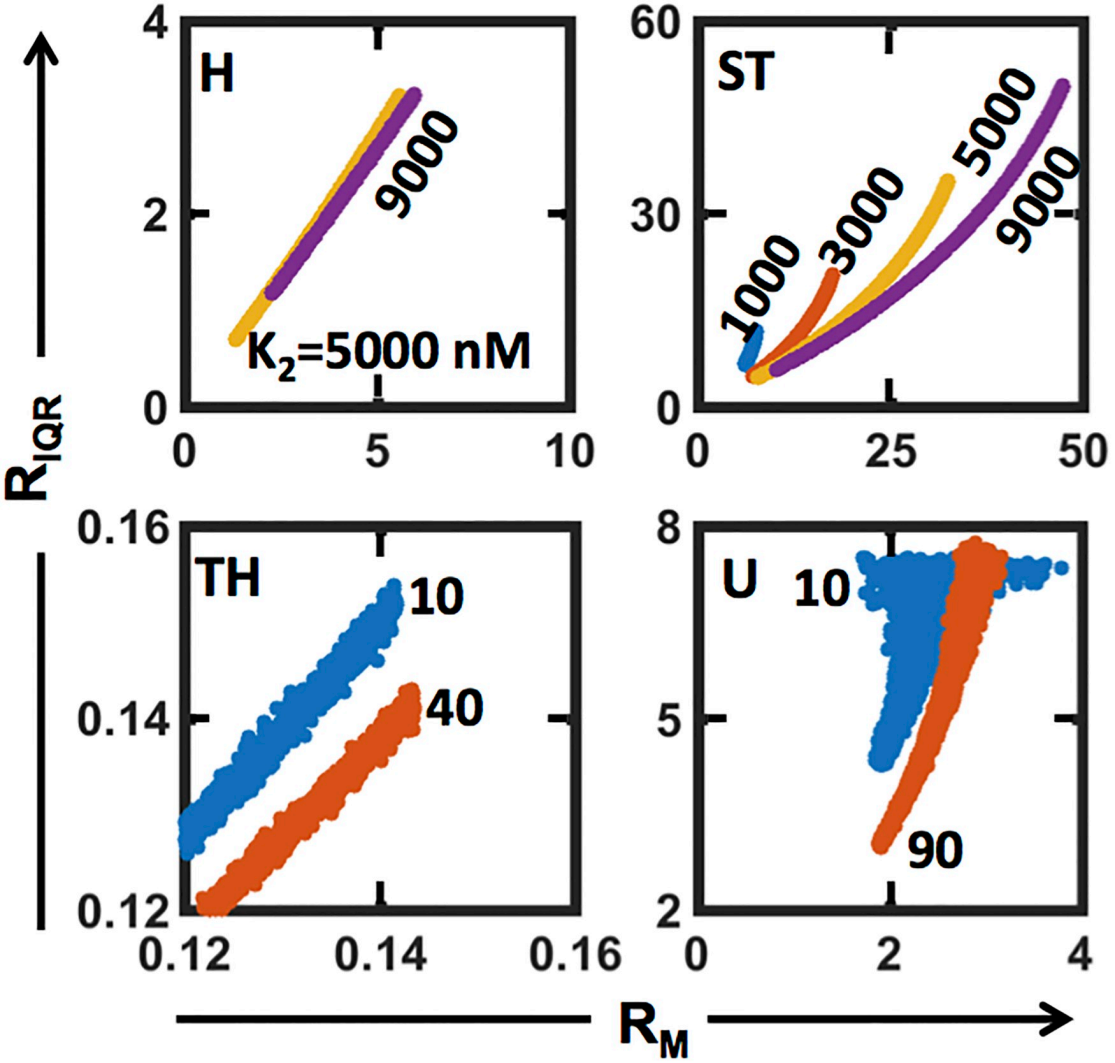
Relationship between R_IQR_ and R_M_ for the regime permitted *K*_*1*_ range at a few fixed *K*_*2*_ values for the four operating regimes. Note that similar relationship for the regime permitted *K*_*2*_ range at a few fixed *K*_*1*_ values is in S7 Fig.

We next assess the relationship between R_IQR_ and R_M_, the ratio of median of pERK (response) and pMEK (input) distributions. Note that R_M_ is also an experimental observable. Moreover, as median of *D*(*E*) is set to *E*_1/2_ of the dose-response curve, its value is a quantitative reflection of chosen *K*_*1*_ and *K*_*2*_ [16] and therefore the median of response distribution is 500nM. In Fig 4, we show, as a scatter, the relationship between R_IQR_ and R_M_ for each of the four regimes. A cluster of scatter points having same color captures (R_IQR_, R_M_) combination for different values of *K*_*1*_ at a fixed *K*_*2*_. On the other hand, clusters having different colors correspond to different fixed *K*_2_. (Note that in S6 Fig, we present the same for a range of *K*_*2*_ with different fixed *K*_*1*_.). In the H regime, the R_IQR_ is linearly correlated to the corresponding R_M_ in the range of *K*_*2*_ (Fig 4-H) or *K*_*1*_ (S6 Fig-H) values. For the case of TH regime (Fig 4-TH, S6 Fig-TH), the linear relationship between R_IQR_ and R_M_ can be observed only after discounting for a small deviation. In the ST regime, while the relationship for a fixed *K*_*2*_ value (Fig 4-ST) is monotonic in the given range of R_M_, the same for fixed *K*_*1*_ value (S6 Fig-ST) is linear. On the contrary, in the U regime (Fig 4-U, S6 Fig-U), there is no correlation as such, which can be attributed to the fact that the variance of response distribution is indeterminate for ultrasensitive profiles (For a detailed proof, see Appendix I). Moreover, the qualitative relationship between the R_IQR_ and R_M_ for H, ST and TH regimes is nearly insensitive to shape parameter variation (S7 Fig). We show in S7 Text that use of log-uniform sampling also predicts the monotonic relationship between R_IQR_ and R_M_ in H, TH and ST regimes in similar ranges of (*K*_1_, *K*_2_).

Using the range permitted by the experimental data (Fig 2) and reported in literature [18], we consider three cases of low, medium and high values for the shape parameter for *D*(*E*). In Table 2, we report the range for the R_IQR_ obtained for the four operating regimes for each of these three cases. Upon discounting for the marginal overlap in the distributions (as shown in S8 Fig), the R_IQR_ (of a given input-output distribution pair) itself can serve as a reasonable marker to identify the regime in which the underlying enzymatic cascade may lie. Note that for high shape parameter, that is, low CV of the input distribution *D*(*E*), R_IQR_ range obtained in the U regime overlaps significantly with that in the ST regime. This could be due to the fact that the underlying dose-response curves in the U regime having R_IQR_ in (~5, ~17.26) are perhaps similar to those obtained in the ST regime but R_IQR_ being in the same range. This suggests that if the input distribution has a shape parameter around the medium or high values reported in Table 3, then R_IQR_ alone may be used to directly infer the operating regime corresponding to the experimentally measured input-output distributions.

**Table 2:** Effect of shape parameter of *D*(*E*) on the lower and upper limits of R_IQR_ for the four regimes.

Regime→ /Shape parameter↓	Threshold Hyperboli R_IQR_	Hyperbolic R_IQR_	Saturated R_IQR_	Signal Transducing R_IQR_
**1.56**	(0.14,0.17)	(0.83,3.24)	(2.55,6.02)	(5.85,54)
**4.35**	(0.12,0.15)	(0.73,2.90)	(2.6,8.03)	(5.20,54.43)
**11.11**	(0.11,0.13)	(0.71,3.01)	(2.66,17.26)	(5.01,54.58)

**Table 3:** R_IQR_, R_M_ and p-Value for Hartigan’s dip test along with likely operating regime that corresponds to the input and response experimental distributions for the three PMA treatment conditions.

Sample	p-value of Hartigan’s dip test	Median ratio (R_M_)	IQR ratio (R_IQR_)	Likely regime
**Control**	0.95±0.005	1.60±0.03	3.08±0.15	Hyperbolic
**1ng/ml PMA**	0.99±0.003	1.93±0.08	3.36±0.007	Hyperbolic
**100ng/ml PMA**	1±0	6.75±0.12	6.78±1.21	Signal-transducing
**1000ng/ml PMA**	0.99±0.001	6.98±0.31	5.43±1.27	Signal-transducing

Since R_IQR_ does not account for the presence of multiple high points in the response distribution, in ST and TH regimes, the R_IQR_ based regime identification can be complemented with the qualitative assessment discussed in the previous section. The qualitative assessment of the response distribution can further aid in refining the (*K*_1_, *K*_2_) parameter space in which the enzymatic cascade may be operating in. Further, the placement of the cascade in the U regime, wherein the response distributions are expected to be bimodal, can be achieved simply by elimination as the monotonic relationship between R_IQR_ and R_M_ cannot be guaranteed. Since R_IQR_ observable is purely based on the properties of snapshot input-response distribution pair, unavailability of the underlying dose-response curve is not an impediment.

### Effect of PMA stimulus on the operating regime

We next use the qualitative assessment and the (monotonic) relationship between R_IQR_ and R_M_ to identify the operating regime that may correspond the steady state pMEK-pERK input-response snapshot distributions in PMA stimulated cells (Fig 2). Hartigan’s dip test show that the pERK response distributions for all three treatment conditions at steady-state (30 mins) exhibit a unimodal behavior with a confidence-level greater than 95% (Table 3) and therefore presence of ultrasensitive behavior is ruled out. (Using Gillespie simulations, we show in S8 Text that the single enzymatic cascade can reach steady state in 30 mins for the parameters based on those in Table 1 and the (*K*_1_, *K*_2_) combinations corresponding to the R_IQR_ range permitted by the snapshot distributions in Fig 2).

R_M_ and R_IQR_ for the control and the three treatment conditions, T1, T2, and T3 are in Table 2. We next assessed the window in the R_M_ vs R_IQR_ space where the experimentally observed (R_M_, R_IQR_) corresponding to pMEK-pERK distribution pair appeared for control and the three treatments. Further we assessed if that window permitted a monotonic relationship between R_IQR_ and R_M_ and then identified the regime corresponding to this window. Note that for each of the comparisons (including that of experimental technical replicates), the model predicted R_IQR_ vs R_M_ space was constructed using the *D*(*E*) that assumed corresponding pMEK distribution guided shape parameter. (R_M_, R_IQR_) values (Table 3) for the resting cells (control) and for those cells exposed to 1 ng/ml (T1) suggest that the underlying regime in both these cases may be operating in the hyperbolic (H) regime. On the other hand, for the other two treatment conditions of 100 ng/ml (T2) and 1000 ng/ml (T3), the corresponding (R_M_, R_IQR_) values suggest that the underlying cascade is likely operating in the signal-transducing (ST) regime.

## Discussion and conclusion

Systems-level understanding of the behavior of a single MAPK enzymatic cascade, a ubiquitously conserved pathway in a cellular signaling network, has been of significant interest due to its capability to strongly modulate the eventual response of a stimulated cell [1–4]. One class of the system-level behavior of the single cascade considered is that of the input-output characteristic or dose-response curve. Using deterministic framework, the dose-response curve has been shown to permit four distinct operating regimes namely hyperbolic (H), signal transducing (ST), threshold hyperbolic (TH) and ultrasensitive (U) behaviors [6,11]. Such a deterministic framework can at best be used for inferring underlying operating regime based on population-averaged experimental data only. Cell-to-cell variability is known to be present in MAPK cascades [8–10]. Population-level measurements using non-plasmid reporter based assays leading to a distribution of abundance of intra-cellular proteins such as pMEK, pERK capture this variability in an ensemble of cells [10]. Such pMEK and pERK distributions are, at best, available only at discrete time points [19]. We show that even in the absence of underlying dose-response curve, the snapshot pMEK and pERK distribution data, the input-response distribution pair of a single MAPK cascade, can be directly used to infer the operating regime at an ensemble-level. Our ability to identify the operating regime based on the snapshot distribution data is primarily due to the *novel* monotonic relationship between the two experimental observables R_IQR_, the ratio of the inter-quartile range of the input-response snapshot distribution pair and R_M_, the median ratio of the pair. We identified the monotonic relationship by systematic analysis of the Michaelis-Menten approximated mechanistic model of the single enzymatic cascade superimposed with an input distribution guided by the experimental snapshot pMEK distribution in Jurkat-T cells stimulated with PMA.

Ability of a single enzymatic cascade to exhibit bimodal response in TH and U regimes at an ensemble-level has been shown before [13,18]. These studies show that simultaneous presence of cell-to-cell variability in both kinase and phosphatase concentrations captured by log-normal/gamma distribution can lead to stochastic bifurcations. In this study, we show that, over a wide range of the Michaelis-Menten constants *K*_1_ and *K*_2_ (Fig 4 and S6 Fig), ST regime may permit bimodal pERK response distribution (Fig 3, S3 and S4 Figs) caused by cell-to-cell variability in *only* pMEK (upstream kinase) abundance. We demonstrate this by using a fitting superposition of the experimental data guided gamma distribution capturing cell-to-cell variability in pMEK, on the dose-response curve permitted by the model. For regimes other than ultrasensitive, Qian et al. suggested that a condition of slow dynamic fluctuations with $\sigma_{K}^{2}+\sigma_{P}^{2}$, sum of variances of distributions of kinase and phosphatase concentrations being large is needed for stochastic bifurcation leading to bimodal behavior [13]. In the ST regime, since only the phosphorylation reaction is saturated, the steady-state pERK level is strongly influenced by the pMEK level. For a nominal CV of 4.35 for pMEK distribution and (*K*_1_, *K*_2_) parameter sets used in Fig 3, the range for variance is (461, 1840). Given $\sigma_{P}^{2}=0$, since $\sigma_{K}^{2}+\sigma_{P}^{2}$ is large, the bimodal behavior exhibited by the model in the ST regime could as well be due to the cell-to-cell variability in the upstream kinase pMEK.

Ability to decipher the operating regime from the experimental snapshot distribution data strongly hinges on the fact that in H, TH, and ST regimes a monotonic relationship between the two observables R_IQR_ and R_M_ exists. This monotonicity relationship could be due to the fact that the unique mapping between the kinase pMEK and phosphorylated protein levels is enforced by the underlying dose-response curve, which may often be unavailable. Our detailed analysis also revealed that no such monotonic relationship exists between the experimental observables in the U regime. This is because the variance in the U regime is indeterminate (Appendix I). R_IQR_, after discounting for overlaps, being well-separated (Table 2) for medium and high shape parameters of the input gamma distribution corroborates the potential use of the monotonic relationship between the R_IQR_ and R_M_ for identifying the operating regime from the experimental snapshot distribution data. It is yet unclear what the relationship between R_IQR_ and R_M_ in the U regime is and why it is not monotonic. While the effect of total ERK protein variability is not considered in this study, it may influence the monotonic relationship observed as the nominal steady-state profiles of the different operating regimes used for categorizing the (*K*_1_, *K*_2_) parameter space is a function of *M*_*t*_, as evident from Eq 4. Thus, a natural extension of this study would be to systematically assess the sensitivity of the monotonic relationship on the other parameters, particularly that of total ERK. The four distinct regimes considered permitted exploration of only ~59% of the (*K*_1_, *K*_2_) parameter space. It is unclear how to characterize the dose-response curves in the remaining ~41% of the (*K*_1_, *K*_2_) space at an ensemble-level. In addition to pMEK, incorporating the cell-to-cell variability in total protein may offer some insights on the potential behavior in this ~41% of the space.

Implementation of the proposed method revealed that, at steady-state, while the cascade in Jurkat E6.1 cells both under resting (unstimulated) conditions and exposed to 1 ng/ml of PMA may operate in H regime, those stimulated with 100 and 1000 ng/ml PMA is likely to have the input-response characteristic of the enzymatic cycle following ST regime. This shift in the operating regime with increase in the stimulus strength could be due to elevated pMEK causing increase in the pERK levels. Thus, the regime that the cascade in stimulated cells depends on the strength of PMA to which they are exposed to. It is yet unclear why, in the cell-line considered, the hyperbolic regime (in unstimulated case) is preserved in cells exposed low dosage of PMA, whereas the signal transduction in cells stimulated with higher concentration of PMA causes a transition to ST regime. Further investigation of the ensemble-level dynamics is needed to assess why, how and when such a transition in the operating regime under steady-state conditions may occur. Understanding this modulation of operating regime by the ensemble-level dynamics could play a strong role in designing synthetic biology based strategies to enable precise, predictive response of the single MAPK enzymatic cascade.

## Materials and methods

### Cell-culture and reagents

Jurkat E6-1 cells were procured from National Centre for Cell Science (NCCS), Pune (India), and cultured in RPMI1640 supplemented with Fetal bovine serum (10%v/v), 2mM/L L-glutamine and 1% antibiotic-antimycotic solution, (procured from HiMedia, India) and were maintained at 37°C in humidified 5% CO_2_. Cells were serum starved overnight prior to harvesting for further use. Phorbol Myristate Acetate (PMA) and Alexa 350, respectively were procured from Sigma Aldrich and ThermoFisher Scientific. Primary conjugated Alexa488-ERK1/2(pT202/pY204) and Alexa647-MEK1/2(pS218) antibodies were procured from BD BioSciences.

### Flow cytometry based single-cell level fluorescence detection

One million harvested cells were stimulated with PMA for the chosen concentrations for 30 min following which cells were fixed using 4% Paraformaldehyde (PFA) for 10 min at 37°C. Cells were then washed and permeabilized using 80% methanol for at least 15min on ice. Stimulated cells were stained and multiplexed using fluorescent cell barcoding (FCB) [25,33] before acquisition on BD FACS Aria. For every exposure time, samples with different treatment concentrations were multiplexed using one color FCB. Besides, the unstimulated replicates were also multiplexed using one color FCB. Captured data was first corrected for morphology variation [34], then deconvoluted and analysed using FlowJo [25]. Details of the method adopted for morphology correction and further data processing are in S2 Text.

### Parameter sampling

Based on the nominal values of (*K*_*1*_, *K*_*2*_) for different operating regimes in Table 1 and uniform distribution, 140000 sets of (*K*_*1*_, *K*_*2*_) were generated using stratified random sampling to represent adequately all four combinations of saturated and unsaturated states of the kinase and phosphatase. Details are in S5 Text.

### Regime identification

For every (*K*_*1*_, *K*_*2*_), and all other parameters fixed, the dose-response curve was generated using the model steady-state mapping (Eq 4) between the upstream kinase (pMEK) and the phosphorylated (pERK) protein. Nominal dose-response curve (profile) was constructed for each regime using the corresponding parameters in Table 1. Relative euclidean distance (*d*_*c*_) between the dose-response curve for a certain (*K*_*1*_, *K*_*2*_) and the nominal profile of the four regimes was estimated. Details are in S4 Text. For a particular (*K*_*1*_, *K*_*2*_), the regime for which *d*_*c*_ < 10%, the dose-response curve corresponding to that parameter combination was earmarked for that regime.

### Hartigan’s dip test

Hartigan’s dip test [31] is based on calculating a dip value which is the maximum distance between the unimodal distribution function and a candidate distribution, and performing a null hypothesis test of the candidate distribution being unimodal. We employ the Hartigan’s test implemented in MATLAB [35].

## Appendix I: Response distribution variance in saturated regime

Based on the stationary distribution of the phosphorylated protein pERK for a single-enzymatic cascade [36], in the ultrasensitive regime, the mean and variance of the distribution, respectively are

$$\eta =\frac{k_{f}E/k_{r}p}{1-k_{f}E/k_{r}p}-(N+1)\frac{{\left(k_{f}E/k_{r}p\right)}^{N+1}}{1-{\left(k_{f}E/k_{r}p\right)}^{N+1}}$$ (AI.1)

and

$$\sigma^{2}=\frac{k_{f}E/k_{r}p}{{\left(1-k_{f}E/k_{r}p\right)}^{2}}-{\left(N+1\right)}^{2}\frac{{\left(k_{f}E/k_{r}p\right)}^{N+1}}{{\left(1-{\left(k_{f}E/k_{r}p\right)}^{N+1}\right)}^{2}}$$ (AI.2)

where, *N* is the ensemble size. All other symbols are as in Eq 3. Using *E*_1/2_ for the (*K*_1_, *K*_2_) samples in saturated regime, the range for *k*_*f*_*E*/*k*_*r*_*p* is [0.9,1]. Thus, for this range, the second term in Eqs AI.1 and AI.2 will vanish for an ensemble size of 20000 considered in this study. As a result, the mean and variance, respectively are

$$\eta \approx \frac{k_{f}E/k_{r}p}{1-k_{f}E/k_{r}p}$$ (AI.3)

and

$$\sigma^{2}\approx \frac{k_{f}E/k_{r}p}{{\left(1-k_{f}E/k_{r}p\right)}^{2}}$$ (AI.4)

For the case nominal profile of saturated regime, wherein both phosphorylation and dephosphorylation reactions are operating under saturation conditions, *k*_*f*_*E* / *k*_*r*_*p* ≈ 1. Thus, the response distribution corresponding to dose-response curves approaching nominal profiles in ultrasensitive regime will have indeterminate standard deviation.

## Supporting information

S1 Fig Two replicates (panels I and II) of the normalized distributions of pMEK and pERK in Jurkat E6.1 cells reported in Fig 2 of main text. (TIFF)

S2 Fig Effect of shape parameter *a* and *K*_*1*_ on pERK distributions on *K*_*1*_ in ST regime for a fixed *K*_*2*_. For the sake of comparison, distributions from Fig 3D (for a = 4.35) are repeated here.
(TIFF)

S3 Fig Effect of shape parameter *a* and *K*_*1*_ on pERK distributions in H regime for a fixed *K*_*2*_. (TIFF)

S4 Fig Effect of shape parameter *a* and *K*_*1*_ on pERK distributions in TH regime for a fixed *K*_*2*_. (TIFF)

S5 Fig Effect of shape parameter *a* and *K*_*1*_ on pERK distributions in U regime for a fixed *K*_*2*_. (TIFF)

S6 Fig Relationship between R_IQR_ and R_M_ for the regime permitted *K*_*2*_ range at a few fixed *K*_*1*_ values for the four operating regimes. Note that similar relationship for the regime permitted *K*_*1*_ range at a few fixed *K*_*2*_ values is in Fig 4.
(TIFF)

S7 Fig Effect of shape parameter on the relationship between R_IQR_ and R_M_. Note that in the ordinate captures the effective slope of the monotonicity between R_IQR_ and R_M_, as estimated using a linear fit.
(TIFF)

S8 Fig Distribution of R_IQR_ (in the form of boxplots) for the four regimes for low, medium and high values of shape parameter *a*. (TIFF)

S1 Text Access to raw data files. (PDF)

S2 Text Morphology correction and data processing. (PDF)

S3 Text Gamma distribution fit of snapshot experimental ensemble data. (PDF)

S4 Text Maximum permitted *M*_*p*_. (PDF)

S5 Text Generation of (K1, K2) samples. (PDF)

S6 Text Estimation of the relative distance between a dose-response curve and nominal profile. (PDF)

S7 Text Monotonicity relationship between RIQR and RM achieved using log-uniform sampling of (K1, K2) parameter sets. (PDF)

S8 Text Stochastic simulations. (PDF)
