# Supplementary figures and images for: Operating regimes in a single enzymatic cascade at ensemble-level
Source: PLoS One. 2019 Aug 1;14(8):e0220243. doi: 10.1371/journal.pone.0220243 (PMC6675077; doi:10.1371/journal.pone.0220243)

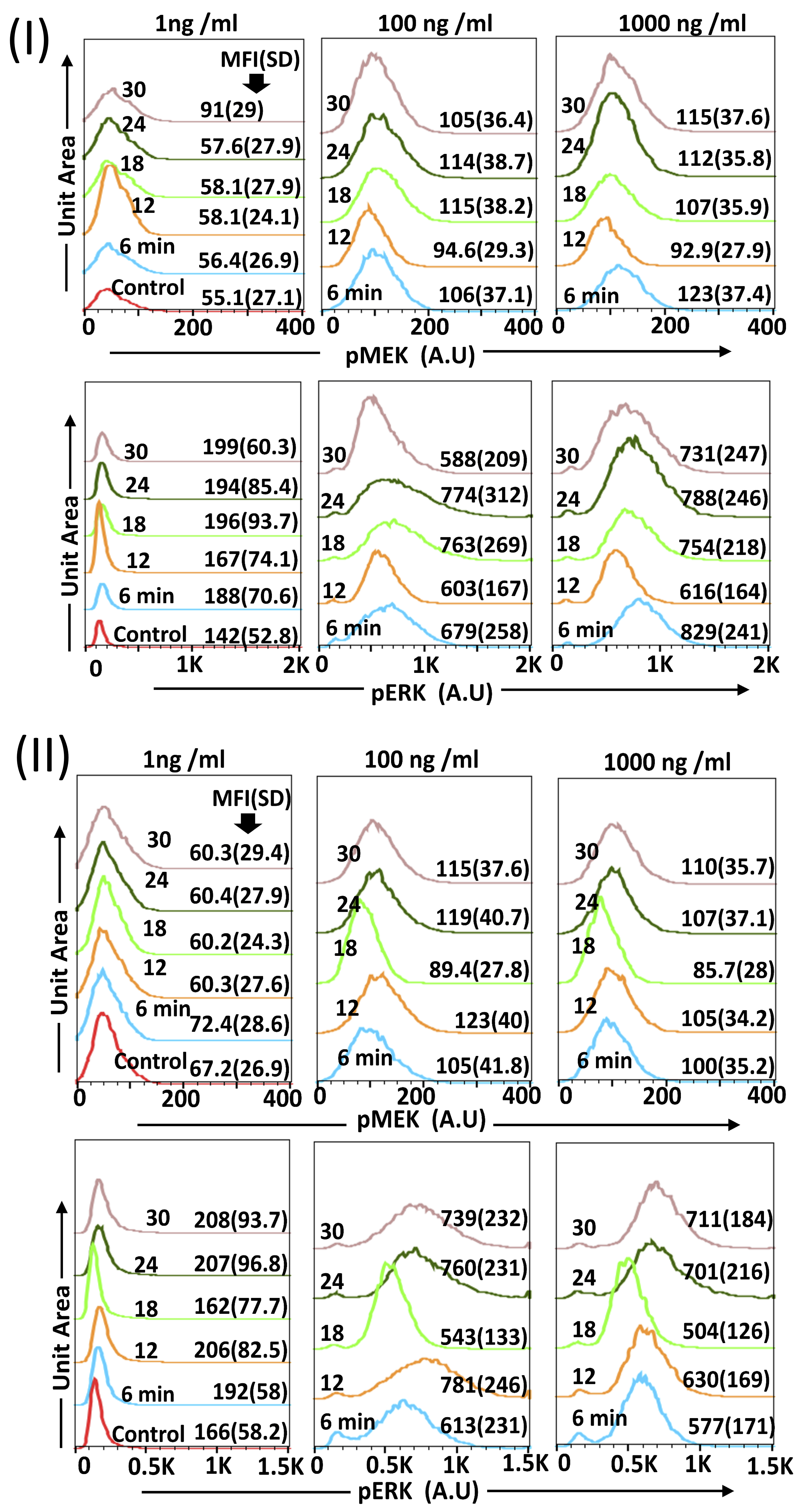

Supplement: S1 Fig — (TIFF) [file pone.0220243.s001.tiff]

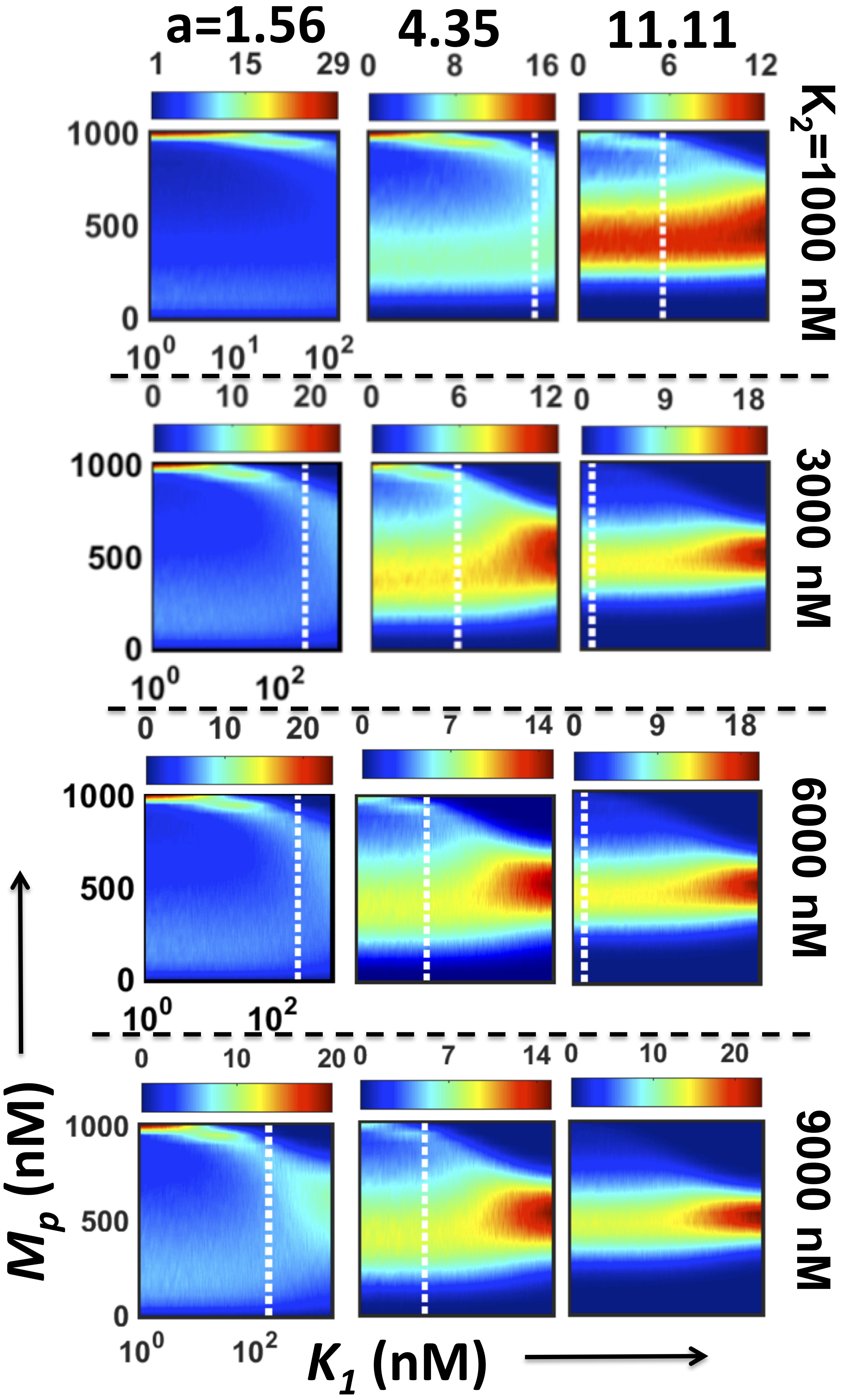

Supplement: S2 Fig — For the sake of comparison, distributions from Fig 3D (for a = 4.35) are repeated here. (TIFF) [file pone.0220243.s002.tiff]

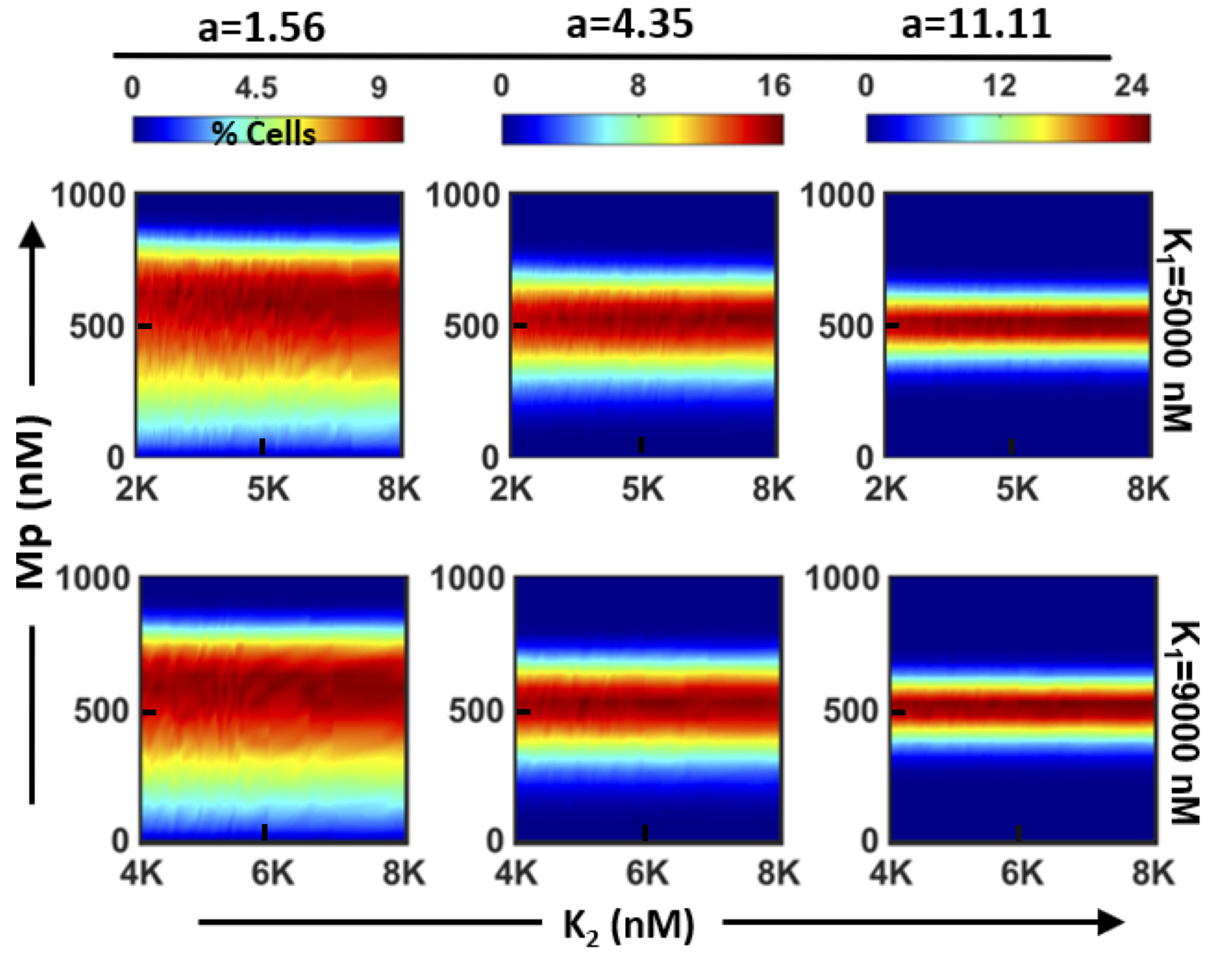

Supplement: S3 Fig — (TIFF) [file pone.0220243.s003.tiff]

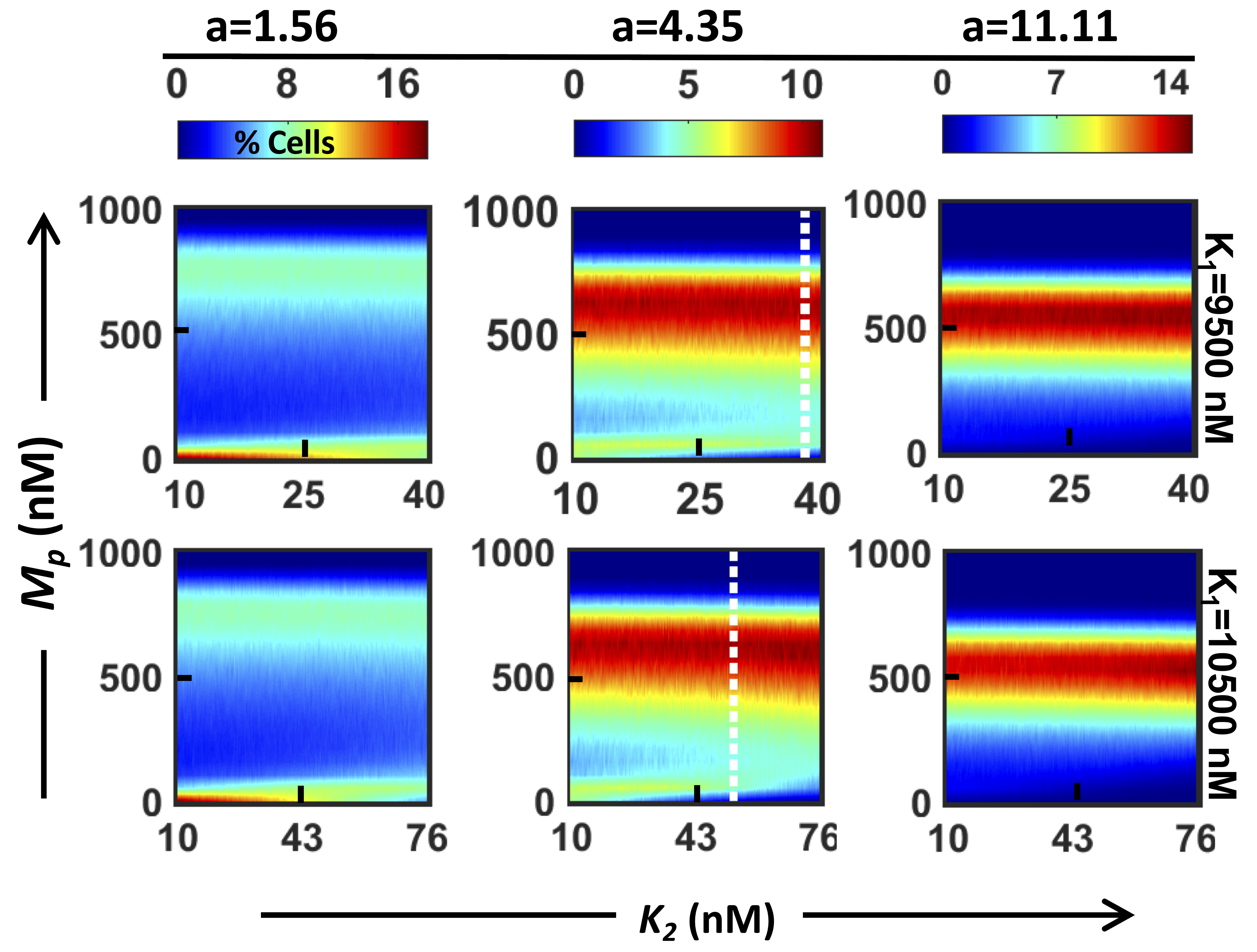

Supplement: S4 Fig — (TIFF) [file pone.0220243.s004.tiff]

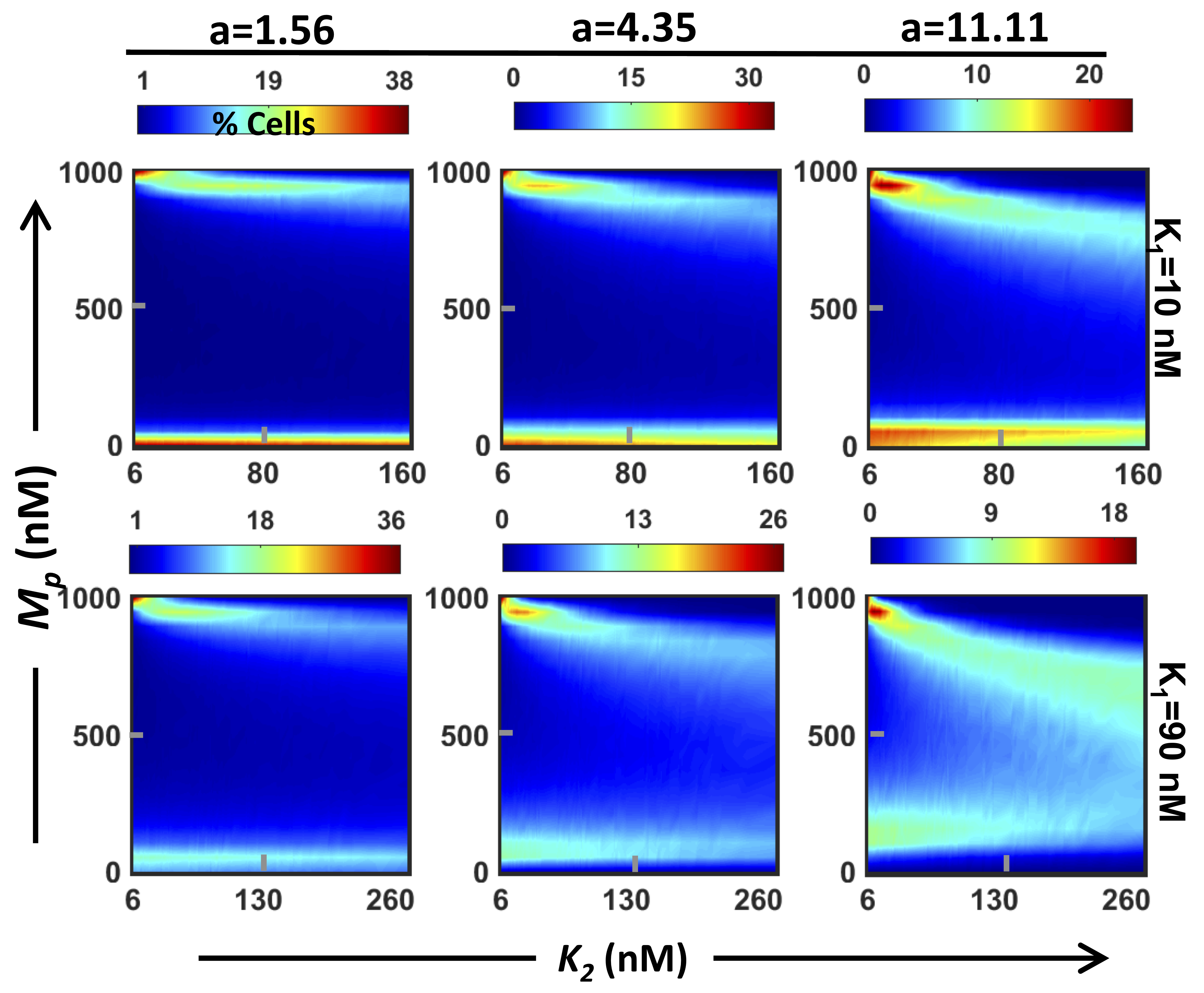

Supplement: S5 Fig — (TIFF) [file pone.0220243.s005.tiff]

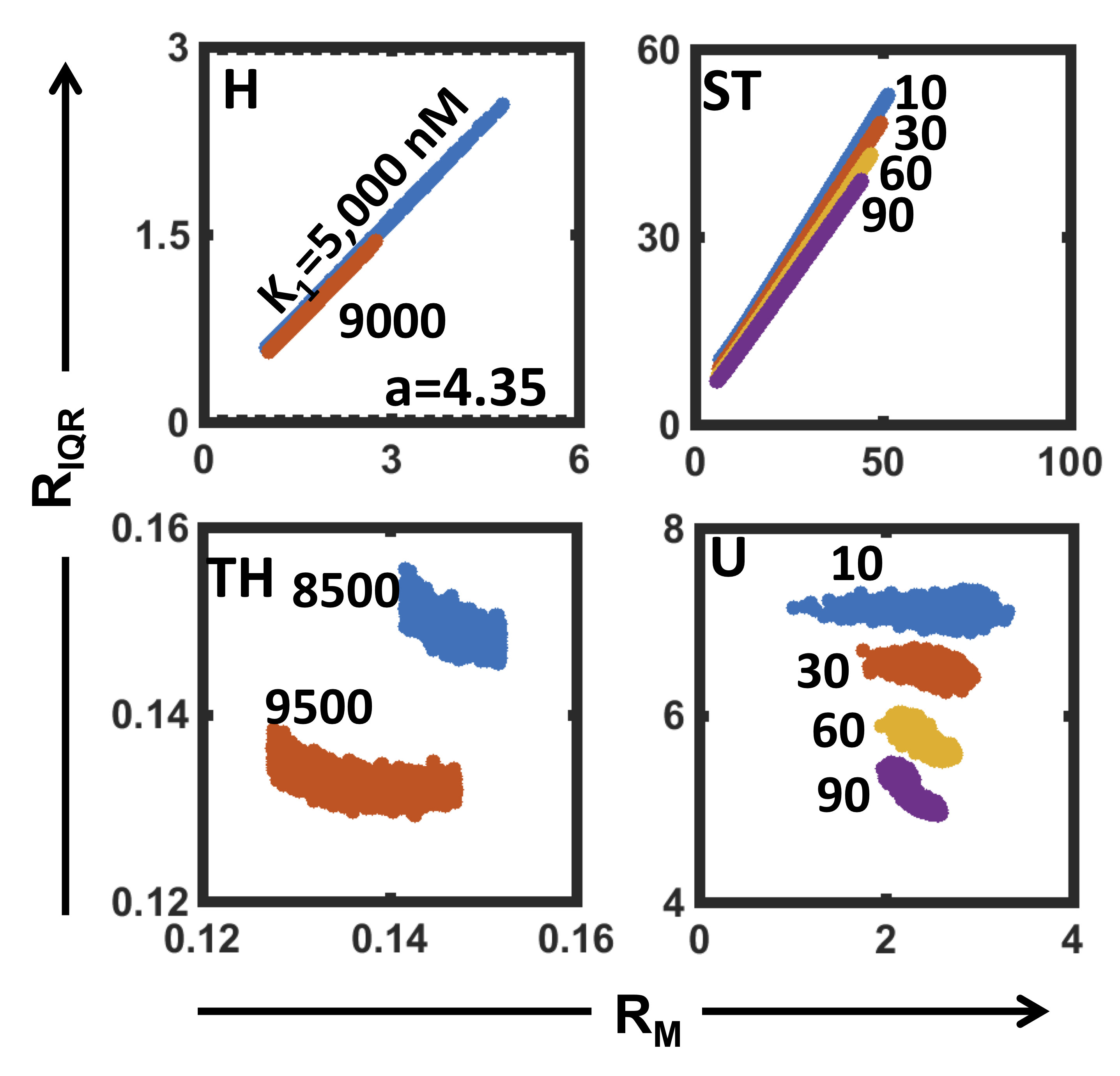

Supplement: S6 Fig — Note that similar relationship for the regime permitted K1 range at a few fixed K2 values is in Fig 4. (TIFF) [file pone.0220243.s006.tiff]

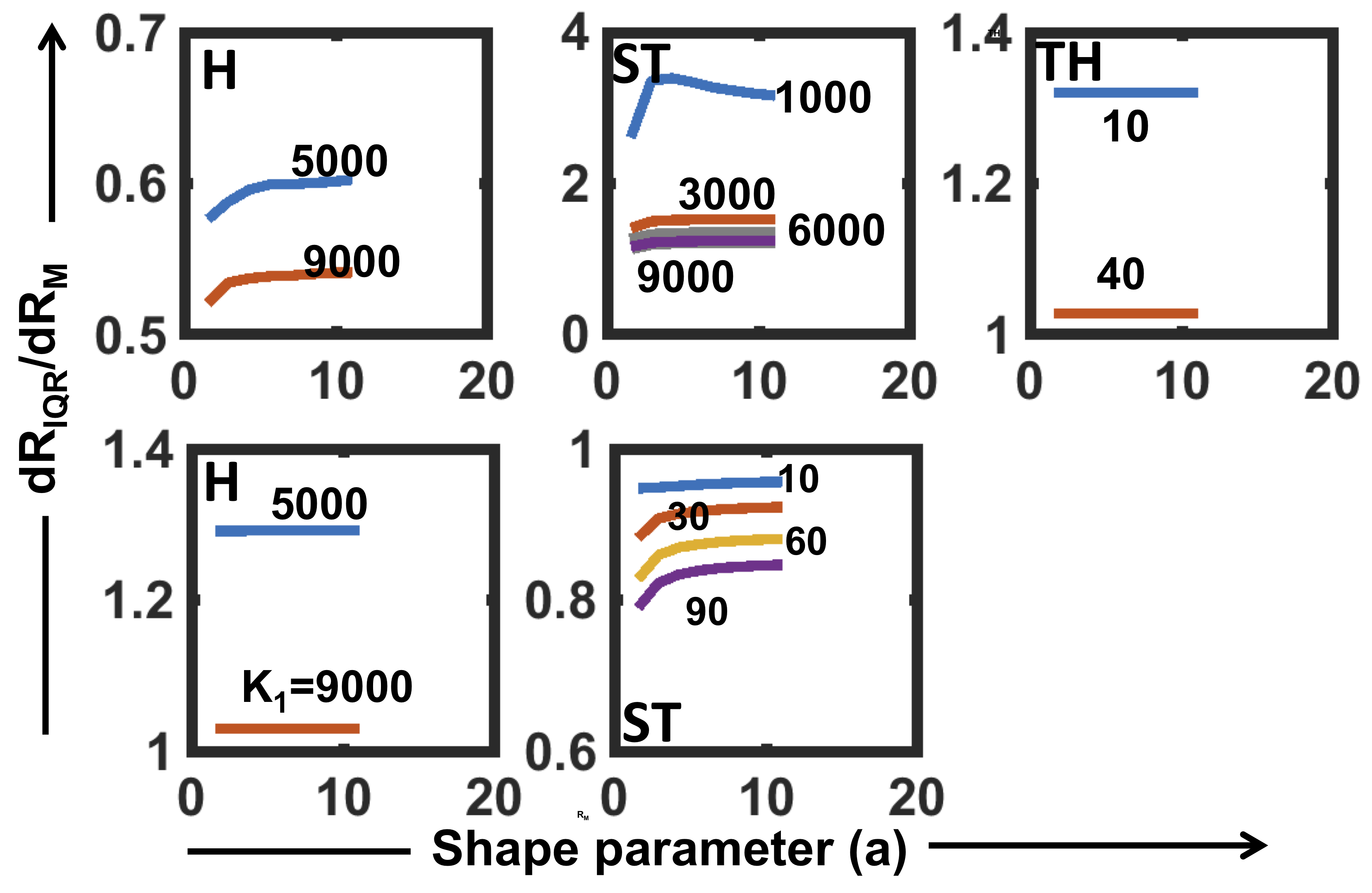

Supplement: S7 Fig — Note that in the ordinate captures the effective slope of the monotonicity between RIQR and RM, as estimated using a linear fit. (TIFF) [file pone.0220243.s007.tiff]

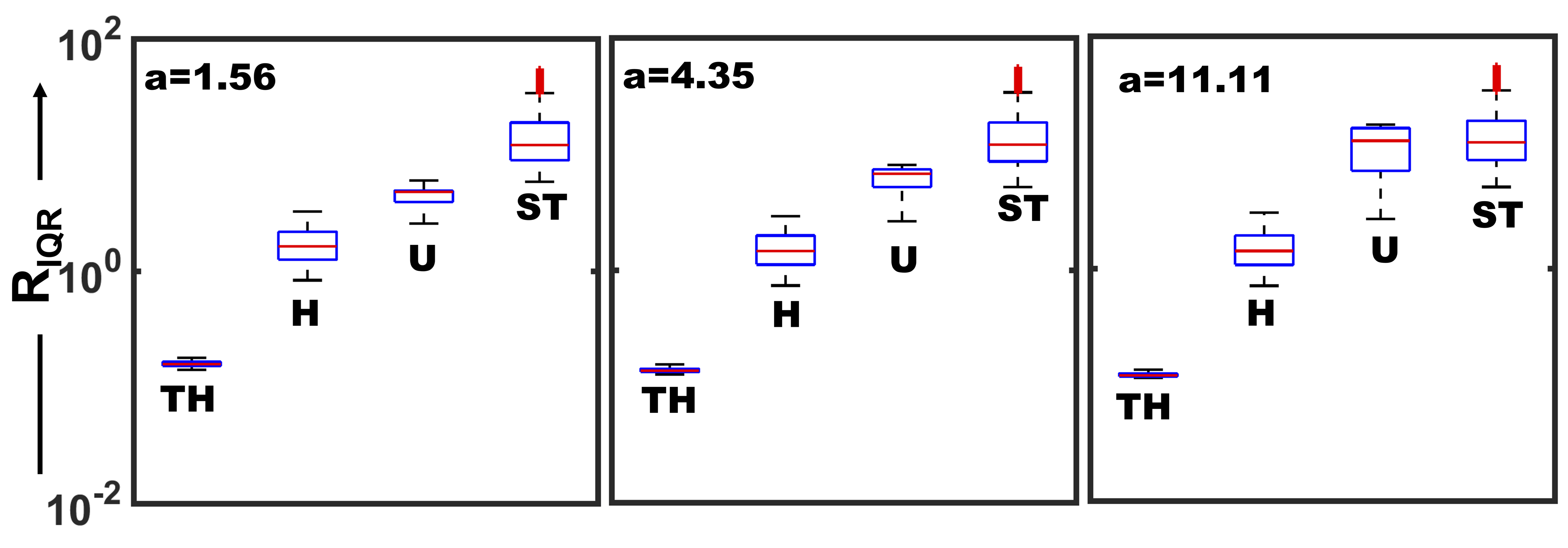

Supplement: S8 Fig — (TIFF) [file pone.0220243.s008.tiff]
