# Supplementary material for: Operating regimes in a single enzymatic cascade at ensemble-level
Source: PLoS One. 2019 Aug 1;14(8):e0220243. doi: 10.1371/journal.pone.0220243 (PMC6675077; doi:10.1371/journal.pone.0220243)
Supplement: S1 Text — (PDF) [file pone.0220243.s009.pdf]

Operating regimes in a single enzymatic cascade at ensemble-level

Supplementary Information

Text S1. Access to raw data files

Akshay Parundekar[1$], Girija Kalantre[1$], Akshada Khadpekar[1], Ganesh A. Viswanathan[1*]

[1] Department of Chemical Engineering, Indian Institute of Technology Bombay, Powai, Mumbai – 400076, India

*Corresponding author
Email: ganeshav@iitb.ac.in

[$]Equal contribution

Access to raw data files

Raw data files corresponding to the experimental results presented in Fig. 2 are available via FlowRepository.org.

**Figure number**	**FlowRepository tracking number (Repository ID)**	**Purpose**	**No. of FCS files**
2	http://flowrepository.org/id/FR-FCM-Z234	To assess the pMEK and pERK levels in an ensemble of Jurkat-T cells stimulated with PMA of certain concentration as a function of time.	19

FlowRepository security code for sharing raw data files
https://flowrepository.org/id/RvFrmMXP8Bba8pLaGPuLNygCU1Y6C7DXLb5oqDnwTQlY8VMZyMSoTGzFod2XwoJe
