# Supplementary material for: Operating regimes in a single enzymatic cascade at ensemble-level
Source: PLoS One. 2019 Aug 1;14(8):e0220243. doi: 10.1371/journal.pone.0220243 (PMC6675077; doi:10.1371/journal.pone.0220243)
Supplement: S2 Text — (PDF) [file pone.0220243.s010.pdf]

Operating regimes in a single enzymatic cascade at ensemble-level

Supplementary Information

Text S2: Morphology correction and data processing

Akshay Parundekar[1$], Girija Kalantre[1$], Akshada Khadpekar[1], Ganesh A. Viswanathan[1]*

[1] Department of Chemical Engineering, Indian Institute of Technology Bombay, Powai, Mumbai – 400076, India

*Corresponding author
Email: ganeshav@iitb.ac.in

[$]Equal contribution

Morphology correction and data processing

The morphology correction is introduced to minimize the variability in fluorescence due to differences in cell size and granularity that may be naturally present, more so as the harvested cells used for experiments are unsynchronized. We employ the method proposed (and the corresponding MATLAB code made available) by Knijnenberg et al. [1] for morphology correction. Briefly, the morphology correction involves first identifying the density of cells in the forward and side scatter, that is, FSC and SSC, two-dimensional space using the bivariate kernel density estimator. Next, a least-square regression model between the fluorescence of every channel (including those for dyes used for multiplexing) and the FSC and SSC is established using the ensemble raw data. A combination of the sample-specific average fluorescence (from the first step) and sample-specific regression model (second step) is used to compute the morphology corrected fluorescence intensities. These sample-specific fluorescence intensities across a population of cells is then subject to further processing using FlowJo® which involved deconvolution of barcoded samples. After morphology correction, the samples were ported into FlowJo® workspace. First, the population of cells were separated (via manual gating) to individual clusters based on the fluorescence intensity in the channel that is used for detecting Alexa 350. Histograms of the individual clusters were created in FlowJo®.

References

1. Knijnenburg TA, Roda O, Wan Y, Nolan GP, Aitchison JD, Shmulevich I. A regression model approach to enable cell morphology correction in high-throughput flow cytometry. Mol Syst Biol. 2011;7:531.
