# Supplementary material for: Operating regimes in a single enzymatic cascade at ensemble-level
Source: PLoS One. 2019 Aug 1;14(8):e0220243. doi: 10.1371/journal.pone.0220243 (PMC6675077; doi:10.1371/journal.pone.0220243)
Supplement: S3 Text — (PDF) [file pone.0220243.s011.pdf]

Operating regimes in a single enzymatic cascade at ensemble-level

Supplementary Information

Text S3: Gamma distribution fit of snapshot experimental ensemble data

Akshay Parundekar[1$], Girija Kalantre[1$], Akshada Khadpekar[1], Ganesh A. Viswanathan[1]*

[1] Department of Chemical Engineering, Indian Institute of Technology Bombay, Powai, Mumbai – 400076, India

*Corresponding author
Email: ganeshav@iitb.ac.in

[$]Equal contribution

Gamma distribution fit of snapshot experimental ensemble data

Based on the observations of Birtwistle et al. [1], we assumed that pMEK normalized histogram (Fig. 2) can be captured by a gamma distribution with probability density function

$$D(E) = \frac{E^{a-1}}{\Gamma(a)b^{a}} exp\left(-\frac{E}{b}\right) \qquad [S3.1]$$

where, E corresponds the pMEK level. a and b are the shape and scale parameters of the distribution, and $\Gamma(a)$ is the gamma function. The mean, variance and coefficient of variance (CV) of the distribution, respectively are given by ab, ab^2 and $1/\sqrt{a}$. Note that shape parameter uniquely specifies the CV. Moreover, as long as a is sufficiently larger than 1, the median of the distribution is equal to $\sim ab\left(\frac{3a-0.8}{3a+0.2}\right)$ from which mean of the distribution ab can be uniquely specified when the median and shape parameter are known [2]. Gamma distribution fit of the experimental steady-state snapshot ensemble data for control and three (30 mins) PMA treatment conditions obtained using Matlab is in Fig. I. Moreover Table I gives the confidence interval for the predicted CV. The fitting was performed using a maximum-likelihood based estimation (implemented in 'fitdist' routine in the 'Distribution Fitting Toolbox' in Matlab R2015b).

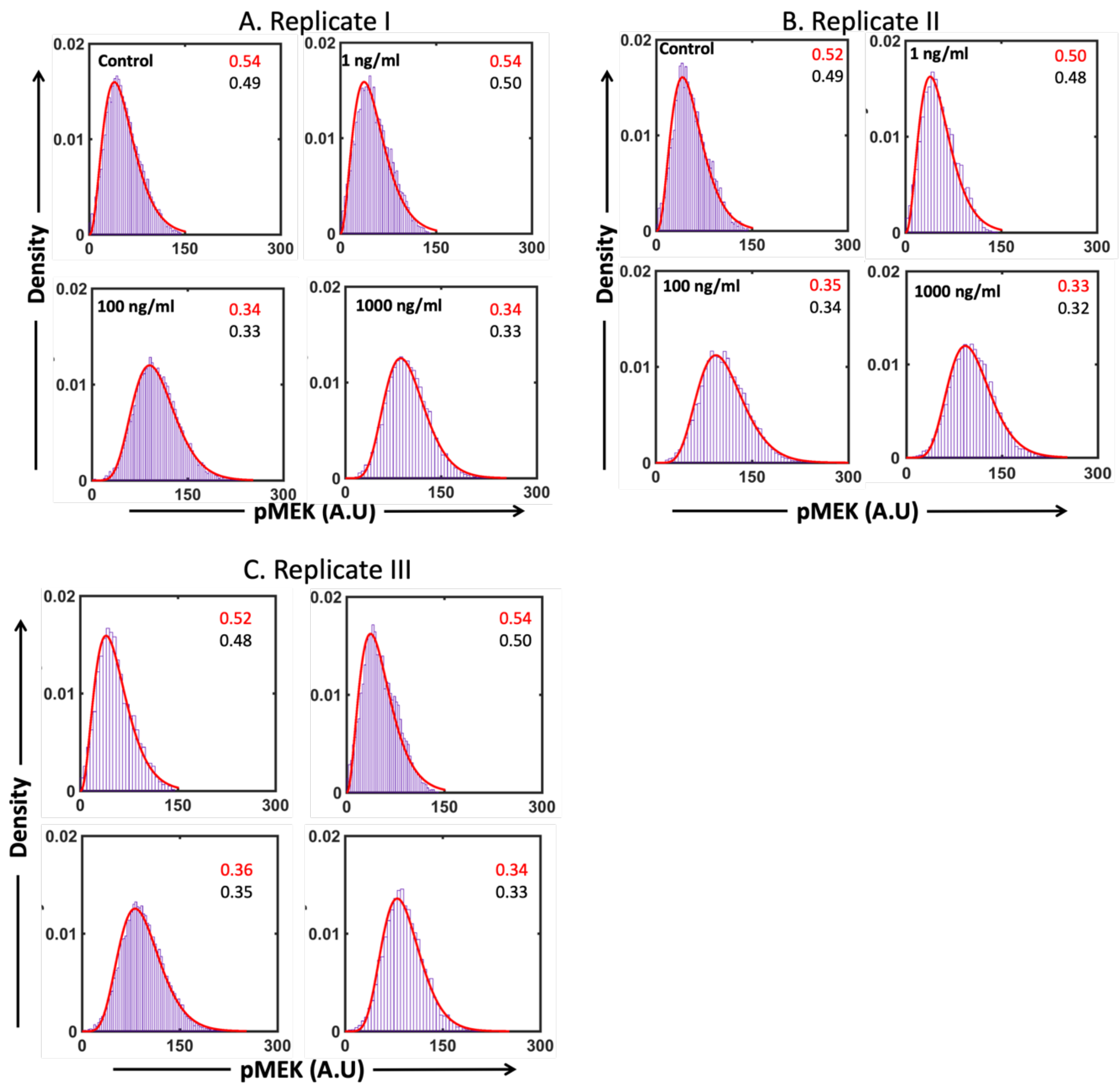

Figure I: Gamma distribution fit (red line) to pMEK ensemble snapshot data (presented in Fig. 1, main text) for control and indicated PMA concentrations for three replicates. Replicates I (A), II (B) and III (C), respectively corresponds to the data in Figs 2, S1-I, and S1-II. The CV of the snapshot data and that of the fit are contrasted along with the fit.

Table I: The confidence interval for CV of all the gamma distribution fitted samples

Replicates/Stimulation	Replicate I	Replicate II	Replicate III
**Control**	[0.51,0.53]	[0.51,0.53]	[0.49,0.51]
**1ng/ml**	[0.51,0.53]	[0.53,0.55]	[0.53,0.55]
**100ng/ml**	[0.34,0.35]	[0.35,0.36]	[0.33,0.34]

**1000ng/ml**	[0.33,0.34]	[0.33,0.34]	[0.33,0.34]

References

1. Birtwistle MR, Rauch J, Kiyatkin A, Aksamitiene E, Dobrzyński M, Hoek JB, Kolch W, Ogunnaike BA, and Kholodenko BN. Emergence of bimodal cell population responses from the interplay between analog single-cell signaling and protein expression noise. BMC Systems Biology 2012;6:109.
2. Banneheka BMSG, Ekanayake GEMUPD. A new point estimator for the median of gamma distribution. Vidyodaya J of Science. 2009; 14: 95-103.
