# Supplementary material for: Operating regimes in a single enzymatic cascade at ensemble-level
Source: PLoS One. 2019 Aug 1;14(8):e0220243. doi: 10.1371/journal.pone.0220243 (PMC6675077; doi:10.1371/journal.pone.0220243)
Supplement: S4 Text — (PDF) [file pone.0220243.s012.pdf]

Operating regimes in a single enzymatic cascade at ensemble-level

Supplementary Information

Text S4: Maximum permitted M_p

Akshay Parundekar[1$], Girija Kalantre[1$], Akshada Khadpekar[1], Ganesh A. Viswanathan[1]*

[1] Department of Chemical Engineering, Indian Institute of Technology Bombay, Powai, Mumbai – 400076, India

*Corresponding author
Email: ganeshav@iitb.ac.in

[$]Equal contribution

Maximum permitted M_p

For a sufficiently large E, Eq. 4 (in the main text) can be reduced to

$$\frac{M_p}{M_t} \approx \frac{\frac{1}{k_r p}\left(1 - \frac{K_2}{M_t}\right) + \sqrt{\frac{1}{(k_r p)^2}\left(\frac{K_2}{M_t} - 1\right)^2 + 4\frac{1}{(k_r p)^2}\frac{K_2}{M_t}}}{\frac{2}{k_r p}} \qquad [S4.1]$$

Simplification of Eq. [S4.1] leads to

$$\frac{M_p}{M_t} = 1 \qquad [S4.2]$$

suggesting that for sufficiently large E, irrespective of the other parameters, all available ERK will transition to the pERK form.
