# Supplementary material for: Operating regimes in a single enzymatic cascade at ensemble-level
Source: PLoS One. 2019 Aug 1;14(8):e0220243. doi: 10.1371/journal.pone.0220243 (PMC6675077; doi:10.1371/journal.pone.0220243)
Supplement: S5 Text — (PDF) [file pone.0220243.s013.pdf]

Operating regimes in a single enzymatic cascade at ensemble-level

Supplementary Information

Text S5: Generation of (K_1, K_2) samples

Akshay Parundekar[1$], Girija Kalantre[1$], Akshada Khadpekar[1], Ganesh A. Viswanathan[1*]

[1] Department of Chemical Engineering, Indian Institute of Technology Bombay, Powai, Mumbai – 400076, India

*Corresponding author
Email: ganeshav@iitb.ac.in

[$]Equal contribution

Generation of (K_1, K_2) samples

The four operating regimes are specified by the saturated or unsaturated state of phosphorylation or dephosphorylation reaction of the cycle. This state can be quantitatively captured by placing bounds on K_1 and K_2 with respect to the total concentration of the substrate corresponding to kinase and phosphatase, respectively [1]. Since the possible range for both K_1 and K_2 is very large (~4 orders of magnitude), we considered a stratified random sampling approach in order to ensure sufficient representation of (K_1, K_2) sets in all the operating regimes. In particular, we ensured (K_1, K_2) samples representing phosphorylation or dephosphorylation or both reaction(s) being saturated is at least 10% greater than those for unsaturated in both the enzymatic reactions of the cycle. The two stratification cut-off points were chosen is such a way that (a) 60000 samples were chosen in the (K_1, K_2) range of [0-1600, 0-1600], and (b) 10000 samples each were chosen in the range [0-50, 0-10000] and [0-10000, 0-50]. In both these cases uniform distribution was used for sampling.

References

1. Gomez-Uribe C, Verghese GC, Mirny LA. Operating regimes of signaling cycles: Statics, dynamics, and noise filtering. PLoS Comp Biol 2007;3: e246.
