# Supplementary material for: Operating regimes in a single enzymatic cascade at ensemble-level
Source: PLoS One. 2019 Aug 1;14(8):e0220243. doi: 10.1371/journal.pone.0220243 (PMC6675077; doi:10.1371/journal.pone.0220243)
Supplement: S6 Text — (PDF) [file pone.0220243.s014.pdf]

Operating regimes in a single enzymatic cascade at ensemble-level

Supplementary Information

Text S6: Estimation of the relative distance between a dose-response curve and nominal profile

Akshay Parundekar[1$], Girija Kalantre[1$], Akshada Khadpekar[1], Ganesh A. Viswanathan[1]*

[1] Department of Chemical Engineering, Indian Institute of Technology Bombay, Powai, Mumbai – 400076, India

*Corresponding author
Email: ganeshav@iitb.ac.in

[$]Equal contribution

Estimation of the relative distance between a dose-response curve and nominal profile

We define relative distance (d_c) between a dose-response curve and nominal profile as

$$d_c = \frac{\left\| \bar{M}_p^c - \bar{M}_p^n \right\|}{\max\limits_{\forall c} \left\| \bar{M}_p^c - \bar{M}_p^n \right\|} \qquad [S6.1]$$

where c represents a dose-response curve for a particular (K_1, K_2), $\bar{M}_p^c$ is the vector of the level of M_p corresponding to a certain E, $\bar{M}_p^n$ is the corresponding vector of M_p for the case of nominal profile of an operating regime. For each of the four operating regimes, we estimated d_c for the dose-response curve corresponding to every (K_1, K_2) sample.
