# Supplementary material for: Operating regimes in a single enzymatic cascade at ensemble-level
Source: PLoS One. 2019 Aug 1;14(8):e0220243. doi: 10.1371/journal.pone.0220243 (PMC6675077; doi:10.1371/journal.pone.0220243)
Supplement: S7 Text — (PDF) [file pone.0220243.s015.pdf]

Operating regimes in a single enzymatic cascade at ensemble-level

Supplementary Information

Text S7: Monotonicity relationship between R_{IQR} and R_M achieved using log-uniform sampling of (K_1, K_2) parameter sets.

Akshay Parundekar[1$], Girija Kalantre[1$], Akshada Khadpekar[1], Ganesh A. Viswanathan[1*]

[1] Department of Chemical Engineering, Indian Institute of Technology Bombay, Powai, Mumbai – 400076, India

*Corresponding author
Email: ganeshav@iitb.ac.in

[$]Equal contribution

Monotonicity relationship between R_{IQR} and R_M achieved using log-uniform sampling of (K_1, K_2) parameter sets.

We generated sufficient samples using log-uniform distribution to obtain at least 15000 (K_1, K_2) parameter sets in every regime. Fig. I shows the joint probability distribution of the generated parameter sets. Using these parameter sets, we repeated the entire analysis to decipher the monotonicity relationship between R_{IQR} and R_M. Fig. II shows the relationship between R_{IQR} and R_M for the regime permitted K_1 range for a few fixed K_2 values for the four regimes. The monotonicity relationship predicted using log-uniform sampling is same as that obtained using stratified uniform sampling shown in Fig. 4.

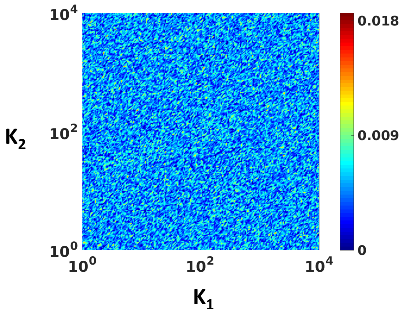

Figure I: Joint probability distribution of the (K_1, K_2) samples generated using the log-uniform sampling.

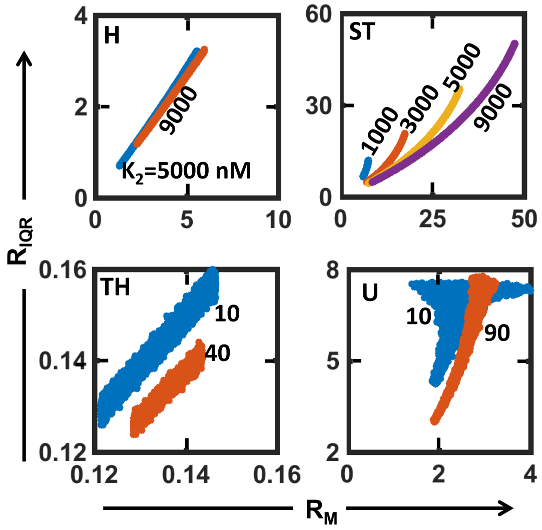

Figure II: Relationship between R_{IQR} and R_M for the regime permitted K_1 range at a few fixed K_2 values for the four operating regimes. Samples were generated using log-uniform distribution (Fig. I).
