# Supplementary material for: Operating regimes in a single enzymatic cascade at ensemble-level
Source: PLoS One. 2019 Aug 1;14(8):e0220243. doi: 10.1371/journal.pone.0220243 (PMC6675077; doi:10.1371/journal.pone.0220243)
Supplement: S8 Text — (PDF) [file pone.0220243.s016.pdf]

Operating regimes in a single enzymatic cascade at ensemble-level

Supplementary Information

Text S8: Stochastic simulations

Akshay Parundekar[1$], Girija Kalantre[1$], Akshada Khadpekar[1], Ganesh A. Viswanathan[1]*

[1] Department of Chemical Engineering, Indian Institute of Technology Bombay, Powai, Mumbai – 400076, India

*Corresponding author
Email: ganeshav@iitb.ac.in

[$]Equal contribution

Stochastic simulations

Using the phosphorylation and dephosphorylation rates in Eq. 1 as the propensity functions for the two enzymatic reaction events [1,2], we performed Gillespie simulations [3] for several (K_1, K_2) combinations whose input-output relationship correspond to the R_{IQR} of the experimental observations. Note that all other parameters were fixed based on those in Table 1 and we converted all relevant parameters/initial concentrations into numbers assuming the cell to be sphere with radius 5.55 μm [4]. For each of the cases, we simulated 100 trajectories. In Fig. I below we show the stochastic dynamics for these trajectories for the parameter set (K_1=658.65 nM, K_2=3000 nM) and the enzyme concentration was set at the corresponding $E_{1/2}$. For this set of parameters, the steady state is achieved at ~25.5 mins and the corresponding mean number of pERK and standard deviation, respectively are 526.1 nM (=2,21,910 molecules) and 1.42 nM. The concentration achieved by solving Eq. 3 for these parameters is 527.5 nM and the standard deviation achieved by superimposition of the pMEK distribution on the input-output curve is given by 151 nM. Note that variability estimated by superimposition of input gamma distribution on the dose-response curve includes cell-to-cell variability embedded in the upstream kinase pMEK level, those from stochastic simulations is primarily due to intrinsic noise in the biochemical reactions. This could explain the differences in the SD estimated using these two approaches. In Table I, we show the time at which steady-state is achieved through stochastic simulations for a few R_{IQR} values corresponding to the experimental distributions in Fig. 2. These suggest that it is possible to achieve steady-state at ~30 mins, as assumed in this study.

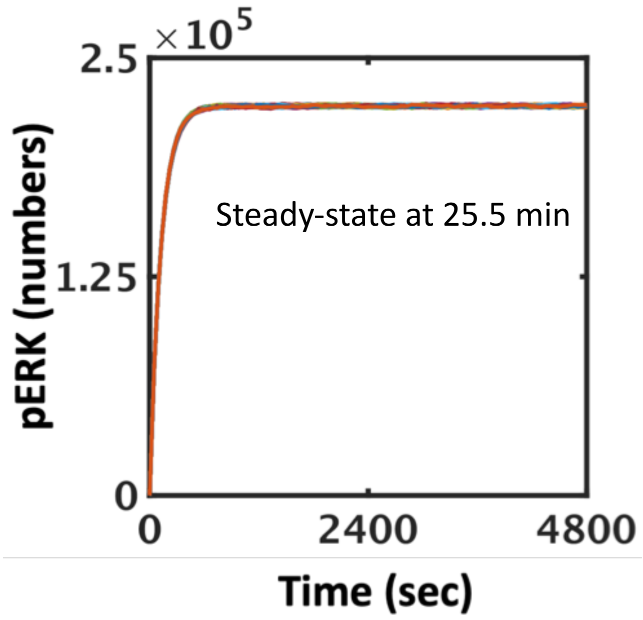

Figure I: Stochastic simulations of the single enzymatic cascade for K_1=658.65 nM and K_2=3000 nM. Mean and standard-deviation, respectively at steady-state are 221910 (=526.1 nM) and 1.42 nM. All other parameters are based on those in Table 1.

Table I: Time at which steady-state is achieved for different R_{IQR} in the range corresponding to experimental data in Fig. 2. Parameters used are based on those in Table 1.

k_r=k_f=0.01				k_r=k_f=0.1			
R_{IQR}	K_1	K_2	Time (min)	R_{IQR}	K_1	K_2	Time (min)
3.04	2055.1	5000	268.88	3.04	2055.1	5000	32.925
3.17	2038.2	5000	269.08	3.17	2038.2	5000	32.949
3.22	4015.9	9000	413.13	3.22	4015.9	9000	50.903
3.23	3621.4	9000	415.41	3.23	3621.4	9000	51.201
3.27	3733.2	9000	414.72	3.27	3733.2	9000	51.112
3.35	1975.8	5000	269.82	3.35	1975.8	5000	33.043
5.62	581.9	3000	215.84	5.62	581.9	3000	26.088
5.72	567.46	3000	216.74	5.72	567.46	3000	26.198

5.79	558.13	3000	217.35	5.79	558.13	3000	26.272
6.27	495.51	3000	221.78	6.27	495.51	3000	26.815
6.69	995.61	6000	335.95	6.69	995.61	6000	41.312
6.75	984.35	6000	336.46	6.75	984.35	6000	41.374
6.83	107.79	1000	160.46	6.83	107.79	1000	19.474
6.89	962.79	6000	337.44	6.89	962.79	6000	41.498
6.94	950.66	6000	338.01	6.9	100.38	1000	19.709
6.98	101.43	1000	162.19	6.94	950.66	6000	41.568
6.99	100.38	1000	162.5	6.98	101.43	1000	19.671

References

1. Rao CV, Arkin A. Stochastic chemical kinetics and quasi-steady-state assumption: Application to the Gillespie Algorithm. J. Chem Phys. 2003; 118:4999-5010.
2. Dhananjaneyulu V, Sagar PVN, Kumar G, Viswanathan GA. Noise propagation in two-step series MAPK cascade. PLoS One. 2012; 7:e35958.
3. Gillespie DT. A general method for numerically simulating the stochastic time evolution of coupled chemical reactions. J Comp Phys 1976; 22:403–434.
4. Milo R, Jorgensen P, Moran U, Weber G, Springer M. BioNumbers—the database of key numbers in molecular and cell biology. Nucl. Acids Res. (2010) 38 (suppl 1): D750-D753.
